# Response of the Carbonate System to Ocean Alkalinity Enhancement: A Mesocosm-Scale Experiment

**DOI:** 10.1021/acsestwater.6c00342

**Published:** 2026-09-26

**Authors:** Selene Varliero, Piero Macchi, Daniela Basso, Paraskevi Pitta, Iordanis Magiopoulos, Chiara Santinelli, Giovanni Checcucci, Silvia Valsecchi, Laura Borromeo, Guido Raos

**Affiliations:** † Department of Chemistry, Materials and Chemical Engineering “G. Natta”, 18981 Politecnico di Milano, Via L. Mancinelli 7, I-20131 Milano, Italy; ‡ Department of Earth and Environmental Sciences, 9305 University of Milano-Bicocca, Piazza della Scienza 4, 20126 Milano, Italy; ¶ Institute of Oceanography, Hellenic Centre for Marine Research, P.O. Box 2214, 71003 Heraklion, Crete, Greece; § CNR-IBF, Istituto di Biofisica, Area della Ricerca, Via Moruzzi 1, I-56124 Pisa, Italy; ∥ Marine and Environmental Sciences Centre (MARE)/Aquatic Research Network (ARNET), Regional Agency for the Development of Research, Technology and Innovation (ARDITI), 9020-105 Funchal, Madeira Island, Portugal; ⊥ Faculty of Life Sciences, University of Madeira, 9000-072 Funchal, Madeira Island, Portugal

**Keywords:** CO_2_ storage, climate change mitigation, marine chemistry, carbonate system

## Abstract

We present the results of a mesocosm-scale experiment conducted in 2023 in the eastern Mediterranean Sea (Crete, Greece) to evaluate the effects of ocean liming via the addition of calcium hydroxide. We assessed two dosage regimes through repeated additions—low (20 μmol L^–1^ alkalinity increase per addition, reaching a total theoretical alkalinity increment of 120 μmol L^–1^) and high (180 μmol L^–1^ per addition, reaching a total theoretical increment of 1080 μmol L^–1^)—comparing against control mesocosms over 14 days, monitoring the pH, total alkalinity, dissolved inorganic carbon, and solid precipitates. The results for the treated mesocosms show that the low dosage effectively increased the total alkalinity and maintained it over the duration of the experiment, unlike the high dosage mesocosms, where the alkalinity dropped within a few days due to the secondary precipitation of carbonates and degassing. In particular, the final net changes of alkalinity were +110 μmol L^–1^ for low dosages (close to the theoretical limit) but amounted to about −330 μmol L^–1^ for the high ones. The concurrent changes in pH were equal to +0.1 and +0.4, while those in dissolved inorganic carbon were equal to −60 and −620 μmol L^–1^, respectively. The fact that, in the low dosage mesocosms, there was a slight reduction in the dissolved inorganic carbon (comparable to that occurring in control mesocosms) despite the total alkalinity increase suggests limited atmospheric CO_2_ uptake. We attribute this to restricted air-sea gas exchange caused by the mesocosm lids, which are commonly adopted in these facilities to prevent the entrance of external contaminants. Our study highlights future directions for testing and enhancing the efficiency of CO_2_ uptake by seawater, improving ocean liming as a viable climate mitigation strategy.

## Graphical abstract

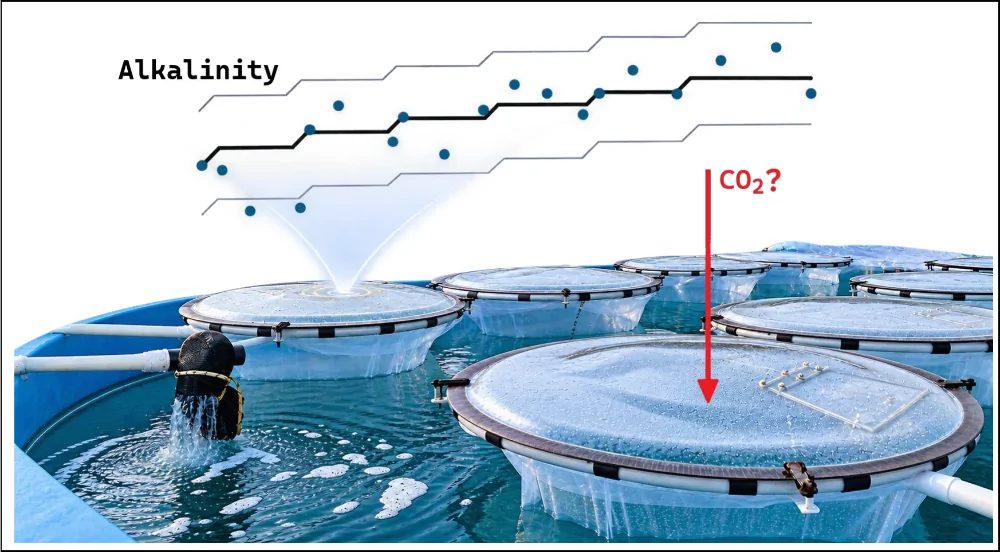

## Introduction

The concentration of atmospheric carbon dioxide (CO_2_) has risen dramatically in the last centuries, from 283 ppm in 1800 to 422 ppm in 2024.^1,2^ While CO_2_ does not have the highest radiative forcing among the greenhouse gases, its reduction represents a priority since it is the most emitted and persistent one over time. In 2019, it accounted for 64% of global CO_2_eq (carbon dioxide equivalent) emissions.^3^

With these premises, keeping the increase of temperature below 2 °C will require the removal of 100 to 1000 GtCO_2_ (= 10^14^–10^15^ kg of CO_2_) from the atmosphere over the course of the 21st century, depending also on the concurrent switch to renewable energies.^4^ These ambitious targets demand the deployment of both carbon dioxide removal (CDR) technologies and effective CO_2_ storage solutions.^3^ Many CDR technologies are currently being developed and tested, but no single technology can fulfill these requirements alone, highlighting the need for a portfolio of solutions.

Ocean alkalinity enhancement (OAE) is a promising method with an estimated CO_2_ storage potential ranging from 1 to 100 GtCO_2_ y^–1^ ^5^ and potential long-term storage for several thousands or even millions of years,^6–8^ although these estimates are largely based on modeling studies. It involves all the species that are part of the carbonate system in seawater: in addition to CO_2_, these include bicarbonate (HCO_3_^–^) and carbonate (CO_3_^2–^) ions (undissociated carbonic acid being negligible).^9,10^ In OAE, CO_2_ is absorbed from the atmosphere and reacts with dissolved alkaline substances, forming CO_3_^2–^ and HCO_3_^–^. This increases both the alkalinity and the dissolved inorganic carbon (DIC) of seawater. Consequently, OAE also contributes to mitigating the ongoing process of ocean acidification by enhancing the oceanic buffer capacity.^11^

OAE was first proposed by Kheshgi, who suggested employing either calcium oxide (CaO) or calcium hydroxide (Ca­(OH)_2_).^12^ When Ca­(OH)_2_ (also known as slaked lime, SL) is used, the process can also be defined as ocean liming (OL). In this OAE application, the release of hydroxide ions increases the pH. This shifts the carbonate equilibrium toward CO_3_^2–^, creating a CO_2_ deficit and inducing atmospheric CO_2_ absorption:

$$Ca{\left(OH\right)}_{2}(s)+2CO_{2}(g)\to {Ca}^{2+}(aq)+2{{HCO}_{3}}^{-}(aq)$$ 1

Afterward, other studies proposed alternative techniques,^13^ including the usage of other minerals such as olivine (Fe,Mg)_2_SiO_4_ ^14^ and electrochemical production of alkalinity,^15^ all of which have both strengths and weaknesses. In this study, we focus on the original implementation of OL through the use of SL.

Following the initial proposals, some experimental work has been carried out to confirm the storage potential of OAEs.^16,17^ In parallel, research is progressing toward modeling the large-scale deployment of OAEs, with a specific outlook on long-term responses of the ocean to the introduction of large amounts of alkaline substances.^18,19^ Ocean liming may also interfere with natural sources of alkalinity, so its effectiveness depends on the location of its deployment.^20^ These studies are paving the way for a comprehensive assessment of the feasibility, scalability, and environmental impacts of OAEs.

OL temporarily raises the local pH of seawater, potentially causing the precipitation of carbonate minerals such as aragonite and calcite (two common polymorphs of CaCO_3_), as well as brucite (Mg­(OH)_2_).^17,21^ This phenomenon was defined as “runaway precipitation”,^16^ and it is undesirable not only due to its unknown effects on marine biota but also because it reduces process efficiency by lowering the alkalinity and releasing some CO_2_ back into the atmosphere.^9,10^ In fact, once triggered by a large spike in pH, precipitation does not stop and may proceed up to a point where the seawater alkalinity is lower than the starting alkalinity, resulting in a negative efficiency.^22^ To mitigate these effects, research focuses on determining optimal dosages of SL that avoid mineral precipitation in transient high-pH, low-dilution scenarios or seeking alternative alkalinization technologies that avoid spikes in pH.^10,23,24^

This paper presents the results of an experiment conducted in 2023 at the Hellenic Centre for Marine Research (HCMR) in Heraklion (Crete, Greece), aimed at investigating OL at a scale beyond laboratory conditions. The objectives and experimental conditions were defined on the basis of a previous experiment, carried out in 2022 in mesocosms at Estación de Ciencias Mariñas de Toralla (ECIMAT) in Vigo (Spain).^25,26^ The two experiments differed in terms of duration, seawater conditions, and dosage levels, allowing us to establish a robust methodology for mesocosm experiments. Both were part of a multidisciplinary effort, involving several research teams. Mesocosms (see Figure 1) were used, as they provide controlled environments that simulate marine systems, avoiding many of the uncertainties associated with open-sea studies. Given that the eastern Mediterranean seawater is oligotrophic, with characteristically low nutrient concentrations,^27^ a key objective of the study was also to assess the biological effects of OL on plankton communities, coccolithophores, and chlorophyll dynamics. Results from the biological experiments have been published elsewhere.^28^ This paper focuses exclusively on the chemical aspects, with particular attention to the carbonate system.

**Figure 1:**
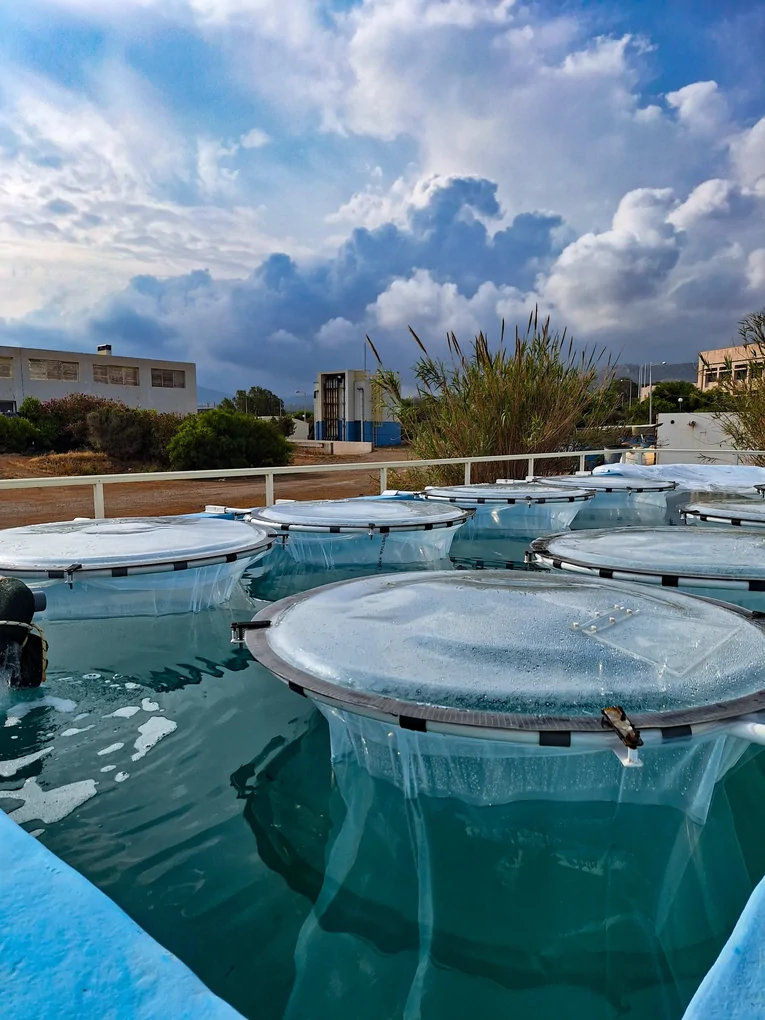
Mesocosm facility at HCMR, Heraklion, Greece (June 2023).

Our experiments aimed to determine the threshold concentration of Ca­(OH)_2_ that can trigger runaway precipitation in OL applications. To investigate a broad range of concentrations and assess both short- and medium-term effects within a single set of experiments, Ca­(OH)_2_ dosages were progressively increased in each mesocosm every 2 days. The experiments were designed to mimic the impacts of SL dissolution in the wake of ships^29^ before the added alkalinity disperses homogeneously on the ocean surface through the action of currents and winds.

## Materials and Methods

### Mesocosm Facilities and Experimental Setup

The experiment used nine land-based mesocosms hosted in large natural seawater pools that stabilized the structures and maintained the temperature constant.

Each mesocosm had a volume of approximately 3 m^3^ and a depth of ∼1.5 m. The exact volume of water was measured during filling on day 0 using a flow meter. Gentle bubbling was maintained for the entire duration of the experiment to ensure good mixing in the water column, avoiding stratification and facilitating equilibration with air. Specific precautions were taken to prevent accidental nutrient contamination of the oligotrophic seawater. As shown in Figure 1, each mesocosm was covered with a lid, allowing limited air exchange while preventing contamination from exogenic matter (e.g., mineral particles carried by wind, leaves, insects, etc.).

Before the first SL addition, a bottle with a funnel on top was placed at the bottom of each mesocosm to collect the possible precipitates. These bottles were retrieved at the end of the experiment.

The main characteristics of the seawater are summarized in Table 1.

**Table 1:** Summary of the Experimental Conditions, Including Average Values and Standard Deviations of Key Seawater Characteristics

Origin of seawater	Eastern Mediterranean
Environment	Oligotrophic
Mesocosm volume at day 0 (m^3^)	3.10
Average temperature (°C)	21.05 ± 0.99
Average salinity (psu)	38.1 ± 2.9
Initial TA (μmol L^–1^)	2740 ± 33
Initial pH	8.20 ± 0.01
Initial DIC (μmol L^–1^)	2456 ± 37

On day 0, the mesocosms were filled with seawater collected close to the HCMR facility. At day 1, the first dosages in the high- and low-concentration mesocosms were applied, and subsequent additions were repeated every 2 days until day 11. Samplings were performed daily at 8:00 AM, while additions were carried out every 2 days, typically 2 h after the morning sampling. On days of addition, an extra sampling was conducted 4 h postaddition.

We performed two types of treatments, which differ by the amount of added Ca­(OH)_2_. Henceforth, we call them high (H) and low (L) dosage treatments. Each of them was replicated in three mesocosms, enabling the testing of a wider range of values while minimizing the risk of uncontrolled or rapid precipitation. Additionally, three mesocosms were not treated with Ca­(OH)_2_ and used as the control (C). These mesocosms, and the samples extracted from them, are labeled H1–H3, L1–L3, and C1–C3, respectively.

Each dosage was administered in six consecutive additions over 2 weeks. The alkalinities added to each mesocosm are reported in Table 2. Note that the values in the table are theoretical; hence, we denote them by the symbol ΔTA_th_. They are equal to twice the nominal concentration of added Ca­(OH)_2_, assuming its complete dissociation and neglecting any side reactions. The highest tested TA dosage was 180 μmol L^–1^, a value determined from preliminary laboratory experiments conducted at HCMR a few days before the start of the mesocosm study. These experiments were performed in 10 L containers using single additions of Ca­(OH)_2_ at different dosage levels, selected on the basis of calculations considering the local seawater chemistry in order to avoid runaway carbonate precipitation. It is important to highlight that the seawater conditions in Crete, characterized by high values of temperature, salinity, and Ca^2+^ concentration, favor calcium carbonate precipitation. Therefore, we chose to apply only modest increases in alkalinity, compared to other studies in the literature.^16,21,22^

**Table 2:** Total Added Alkalinity (ΔTA_th_) Reached at Each Day in the L and H Mesocosms, in μmol L^–1^

Addition no.	Day	ΔTA_th_ (H)	ΔTA_th_ (L)
1	1	180	20
2	3	360	40
3	5	540	60
4	7	720	80
5	9	900	100
6	11	1080	120

Ca­(OH)_2_ was kindly provided by Unicalce with a declared purity of 93.96%. It was added in the form of a 1.5 M slurry, prepared with the water of each mesocosm collected during the morning sampling on the same day. Calcium hydroxide was weighed in at the proper amount to be added to each mesocosm and adjusted to account for their different volumes. The slurry was homogenized by mixing seawater and Ca­(OH)_2_ in a capped glass bottle and vigorously shaking it by hand for approximately 1 min. Immediately after pouring, the contents of the mesocosms were mixed by drastically increasing air bubbling at the surface for about 1 min.

### Sample Handling and Measurements

Water sampling from the mesocosms was performed by using a simple low-disturbance system. From the beginning of the experiment, each mesocosm was equipped with a small sampling tube permanently installed in the water column, positioned approximately at mid-depth. Samples were collected by applying suction with a syringe, allowing seawater to be withdrawn without disturbing the mesocosm environment, and transferred directly into polycarbonate bottles. To minimize contamination, each mesocosm was assigned a dedicated syringe. In addition, the first 30 s of flow was discarded to avoid collecting stagnant water inside the sampling tube, ensuring that only the circulating mesocosm water was sampled.

After the sampling, the water was filtered through a 0.2 μm poly­(ether sulfone) membrane. A first set of measurements took place on site, while part of the samples were stored at 4 °C in glass bottles and transported inside a cooler to Politecnico di Milano for subsequent analyses.

#### On-Field Measurements

At HCMR, the pH was monitored both with field probes and with the bench instrument Thermo Scientific Orion Star A111 pH meter. The pH was measured both before and after filtration. The pH probe was calibrated according to the NIST scale, and the recorded values were then converted to the total scale,^30^ accounting also for minor temperature differences between the samples. All pH values reported in this paper are expressed on the total scale. The measured conductivity was used to calculate the practical salinity.^31^ The total alkalinity was measured by automatic titration (Hanna Instruments HI84531).

#### Laboratory Measurements

At the chemistry laboratory of Politecnico di Milano, pH and conductivity were measured using SevenExcellence electrode sensors (METTLER TOLEDO), applying the same calibration methodology as that used for the field measurements. TA was determined by using the same titration procedure adopted on site. The duplicate measurements of pH and TA performed both in the field and in the laboratory were used to assess possible contamination or sample degradation during storage and transportation. Dissolved inorganic carbon (DIC) could not be measured at HCMR and was measured at Politecnico di Milano by acidification followed by nondispersive infrared detection of produced gaseous CO_2_ using an Analytik Jena multi N/C 2100S analyzer.^32^

The solid particles collected at the end of the experiments were dried and analyzed by X-ray powder diffraction (XRD) using a Bruker D2-phaser diffractometer at Politecnico di Milano. Raman spectra were collected by a Horiba Jobin Yvon LabRAM HR Evolution spectrometer. The system is coupled to an Olympus BXFM petrographic microscope that works in transmitted and reflected light and is connected to a 5Mpx camera (objectives: 5× and 10×; LWD: 50× and 100×; spot size: ∼1 μm). The laser in use was a green Nd laser (wavelength: 532 nm), characterized by a focal length of 800 mm and two grids (600 grooves/μm) and a CCD detector (1024 × 256 px, −60 °C). Most of the 9 filters (100%, 50%, 25%, 10%, 5%, 3%, 1%, 0.1%, and 0.01%) were used to diminish laser power, along with EDGE filter. Around 300 Raman spectra were randomly collected directly on the precipitate mounted on a microscope slide. We collected 28 to 41 spectra from each of the three mesocosms for each treatment (C, L, H) and treated them with LabSpec 5 software in order to identify the mineral phases.

### Data Analysis

The pH, DIC, and TA data were analyzed to assess the quality of the measurements, monitor the stability of the mesocosms, and calculate the aragonite saturation state (Ω_ar_) and the equilibrium partial pressure of CO_2_ (pCO_2_).

We applied statistical inference using control charts like those used in quality analysis of industrial processes.^33^ Standard deviation charts, calculated from the three samplings of mesocosms at each time, were used to identify possible outliers in the measurements. Charts of the sample averages were instead used to identify the possible out-of-control status of the mesocosms upon the addition of Ca­(OH)_2_. For the constructions of the charts, the population standard deviations were calculated from the three control mesocosms, whereas the expected values (specification values) of pH, TA, and DIC were obtained from simulations of the mesocosms using the CO2SYS software,^34^ given the known additions of Ca­(OH)_2_. The comparisons between the experimental values and the predictions clearly indicated that the re-equilibration of CO_2_ with the atmosphere had not taken place (see below). Therefore, the control charts were calculated assuming mesocosms nonequilibrated with the atmosphere, i.e., with a constant DIC.

The averaged pH and TA data were used to calculate the evolution of Ω_ar_ and pCO_2_ in the system. The data set, consisting of cleaned and averaged values, was analyzed using the CO2SYS Python library.^34^ The software used constants from Lueker et al.^35^ for carbonic acid dissociation, Dickson^36^ for KHSO_4_, and Uppström^37^ for the total boron content (B_T_). The calculation of Ω_ar_ in CO2SYS does not consider the variation of Ca^2+^ due to the dissolution of Ca­(OH)_2_ and precipitation of CaCO_3_, so the value was corrected as suggested by Moras et al.:^16^

$${\left[{Ca}^{2+}\right]}_{corr}={\left[{Ca}^{2+}\right]}_{salinity}+\frac{\Delta TA}{2}$$

where ${\left[{Ca}^{2+}\right]}_{salinity}$ is the calcium concentration calculated by CO2SYS from the salinity.

## Results and Discussion

Before diving into the discussion of the results, let us consider briefly the reliability and reproducibility of the experimental measurements. The standard deviation charts reported in the Supporting Information (SI) indicate good reproducibility among the three mesocosms within each treatment. Only a small number of measurements were identified as unreliable, flagging 7 outliers out of the 120 measurements of pH, DIC, and TA performed after the sample delivery for the L and H treatments. Figure 2 illustrates the differences in TA and pH, as measured in the field at HCMR immediately after sampling and filtration or delivered and subsequently analyzed in the laboratory at Politecnico di Milano. A decrease in pH and an increase in TA (3.35% on average) are observed in the latter. The simultaneous occurrence of these two phenomena is unusual, suggesting that they derive from separate processes. The pH decrease is likely due to CO_2_ adsorption, possibly resulting from sample handling in different environments. On the other hand, the TA increase also suggests a rise in DIC but likely in the form of carbonate and bicarbonate ions. The alkalinities calculated from the pH and DIC were systematically higher (by 200–300 μmol L^–1^) than those measured in the laboratory (see the Supporting Information). These differences are not negligible, and we hope to investigate their origin in future experiments. Nonetheless, these effects were consistent across all mesocosms, with no preferential treatment bias. The fact that both L and H treatments behaved similarly to the control samples reinforces the hypothesis that these changes are due to external factors. Consequently, all samples were deemed reliable and analyzed in Milano, where DIC measurements were feasible.

**Figure 2:**
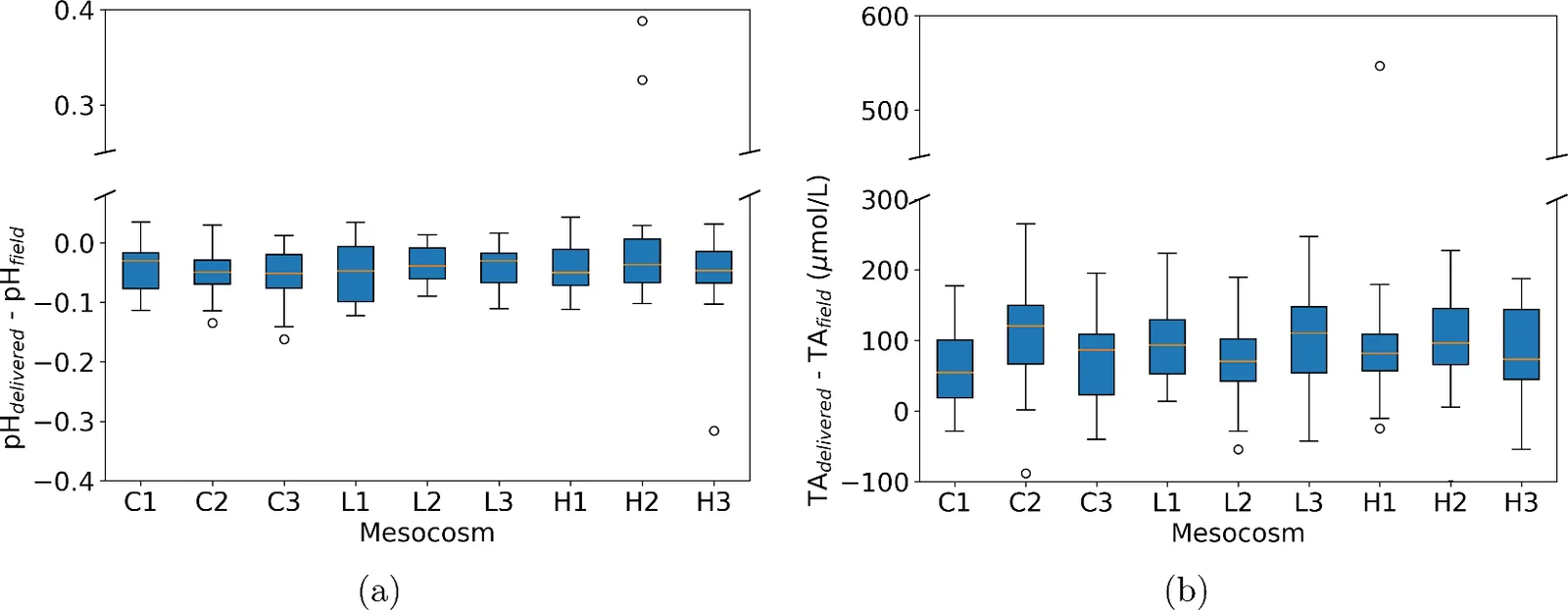
Differences in (a) pH and (b) TA in the samples measured in the field and those delivered to Milano. The blue boxplot extends from the first to the third quartile. The orange line represents the median, and white circles represent outliers.

Figure 3 presents the parameters measured in the laboratory at Politecnico di Milano. First of all, we observe that even the control mesocosms show variations over the time of the experiment, in particular a decrease in TA on days 2 and 6. This is remarkable, considering that the laboratory measurements tend to overestimate the TA by about 100 μmol L^–1^, compared to those obtained in the field (see again Figure 2). Nonetheless, the L treatment closely follows the behavior of the control mesocosms and, unlike the H treatment, retains higher TA levels without any significant loss on the day after each addition. This also applies to the other measurements. In the L treatment, no significant variation of DIC was observed relative to the control, whereas in the H treatment, DIC displayed a strong decrease beginning on day 6, after the fourth addition.

**Figure 3:**
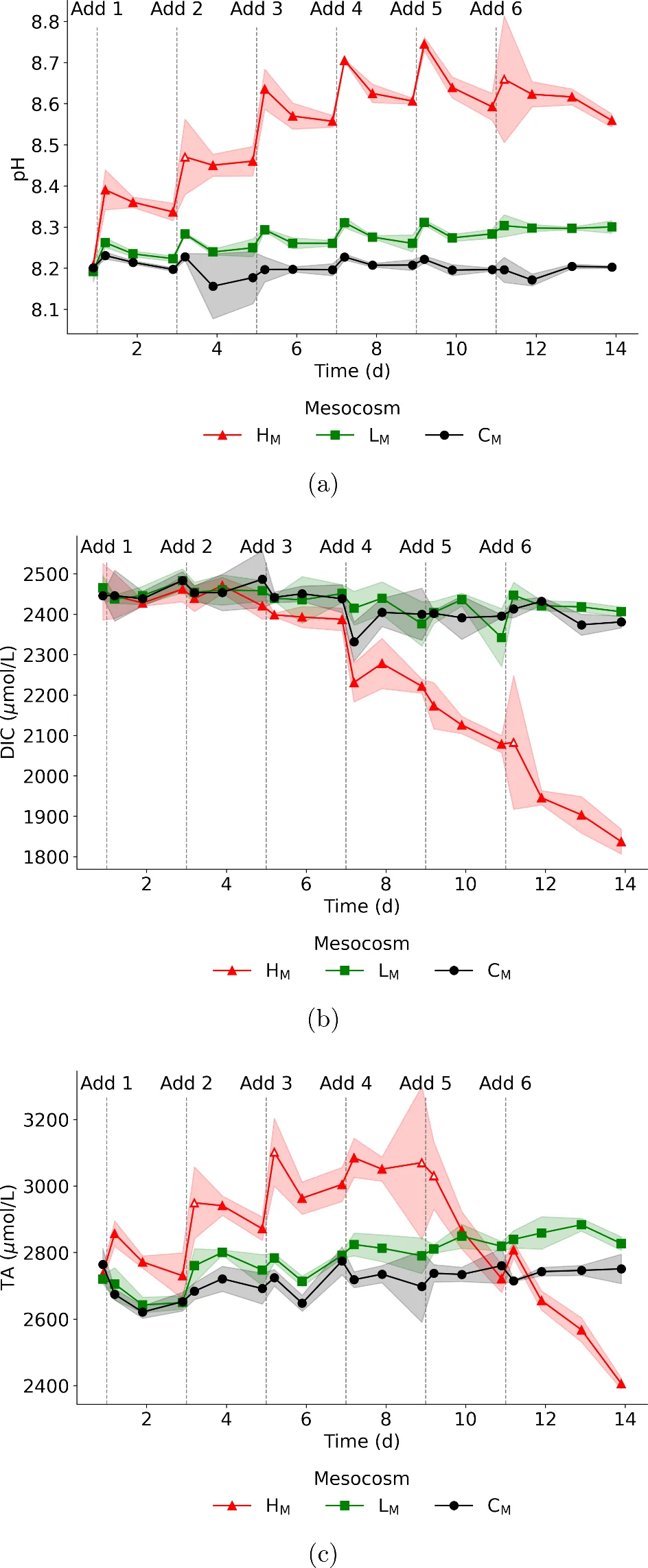
(a) pH, (b) DIC, and (c) TA evolution, measured in Milano on samples from the high (H_M_), low (L_M_), and control (C_M_) mesocosms. The lines are averages of the three replicates, and the colored areas represent the standard deviations. White symbols indicate time points with elevated standard deviation of the mean, as defined in the Materials and Methods section. Vertical lines signal the additions of Ca­(OH)_2_.

The loss of stability in the H treatment is also evident in Figure 4, which compares the temporal evolution of the measured TA with the expected increase following alkalinity additions (see the SI for the other parameters). In the L treatment, the mean TA values remain within the control limits for most of the experimental period, indicating stable system behavior. In contrast, the mean TA values in the H mesocosms progressively deviate from the control limits after day 3, falling outside the error range and indicating progressive destabilization of the systems.

**Figure 4:**
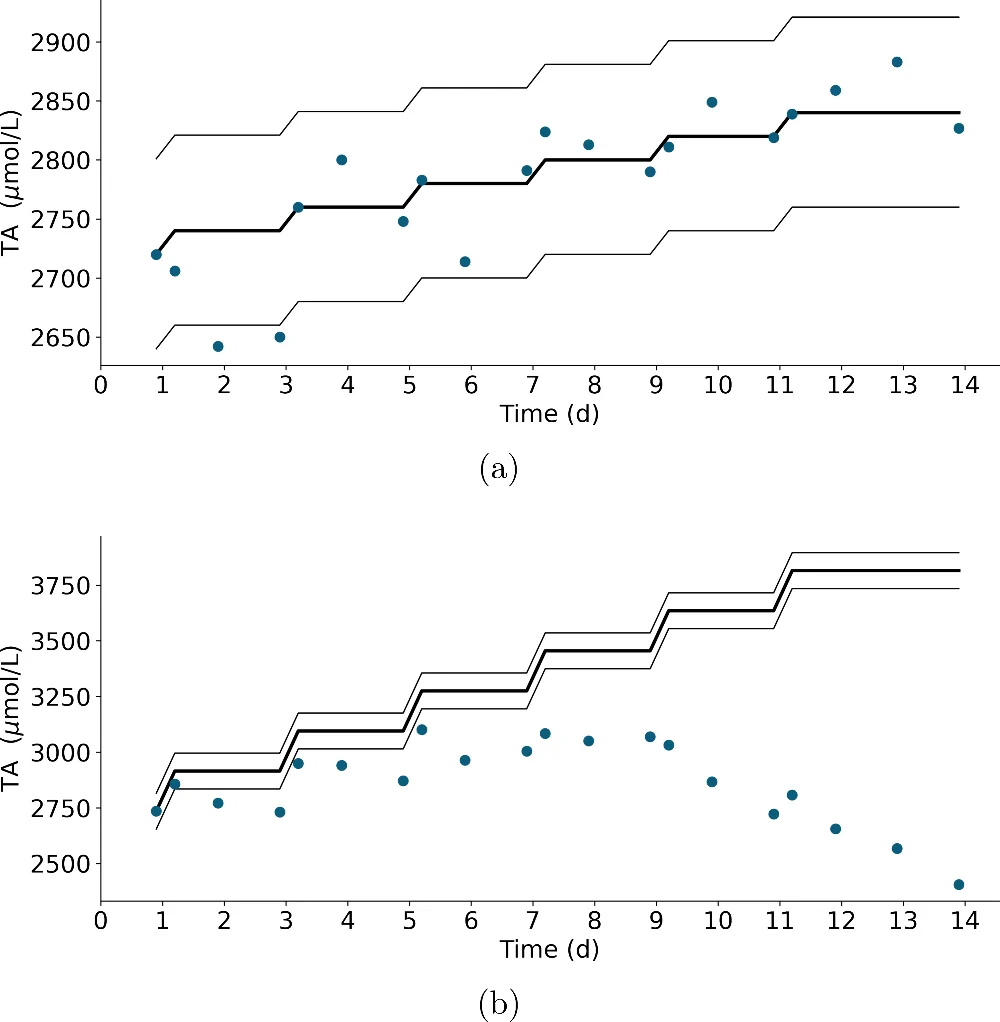
Control charts of sample averages for the (a) L and (b) H mesocosms, showing the temporal evolution of the sample mean of TA values during the experiment. The central line is the bold solid line, while the thin lines indicate the upper and lower control limits (modified by the additions). The dots represent the observed sample means.

Both TA and DIC are key parameters for the marine carbon system. An increase in DIC indicates an uptake of atmospheric CO_2_, while TA is essential for detecting CaCO_3_ precipitation, as it directly reflects the disappearance of CO_3_^2–^ from the solution.^9^ In this experiment, precipitation was visually observed in both H and L mesocosms from the first addition, though to different extents. In fact, some precipitation also occurred in the C mesocosms. To assess this quantitatively, the increase in TA relative to the control was calculated as follows:

2 $$\Delta TA={TA}_{X,i}-{TA}_{C,i}$$

where *X* refers to treatment type (L or H) and *i* denotes the sampling time (in days). A similar equation was applied to calculate ΔDIC, assuming that each mole of Ca­(OH)_2_ added can theoretically uptake 2 mol of CO_2_. The results for both parameters are presented in Figure 5.

**Figure 5:**
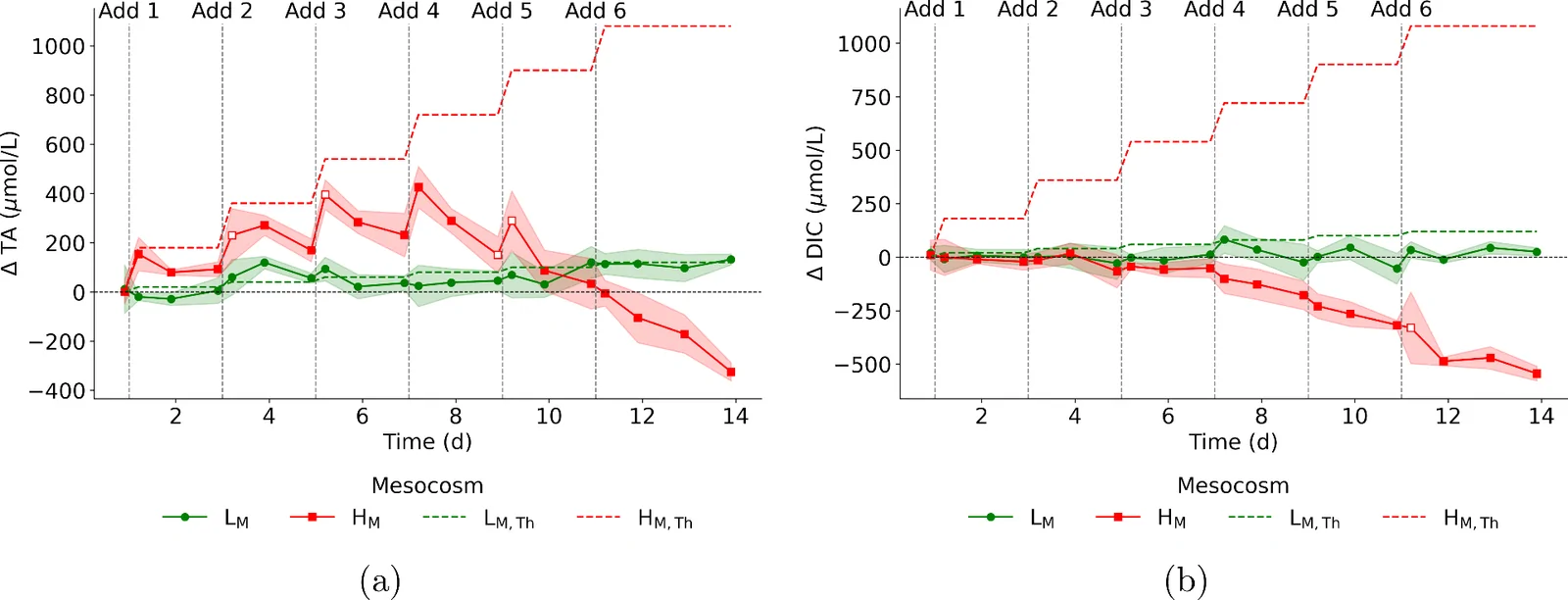
Evolution of (a) ΔTA and (b) ΔDIC. White symbols indicate points with large standard deviation of the mean. Vertical lines signal the additions of Ca­(OH)_2_, while the dashed red and green lines represent the theoretical values that ΔTA_th_ and ΔDIC_th_ should reach according to the treatment.

In the H treatment, TA reaches the expected increase 4 h after each addition but declines on the following day, indicating the precipitation of carbonates. Some precipitation was observed already after the first addition, but it initially involved only a minor portion of added TA.

The H treatment exhibits a significant decrease in ΔDIC, most likely due to CaCO_3_ precipitation, which removes both DIC and TA from the solution. In contrast, DIC levels in the L treatment remain stable, implying very limited precipitation but also little or no absorption of atmospheric CO_2_. This behavior could be attributed to inefficient air exchange between the mesocosms and the atmosphere, likely caused by the small surface area available for gas exchange compared to the large volume of the mesocosms.

The lids placed on top of the mesocosms may have affected the CO_2_ equilibration efficiency. This factor was not considered ahead of the experiments, as previous studies conducted at the HCMR facility showed stable oxygen levels in the mesocosms, and therefore, gas exchange was not expected to be significantly affected. However, it is important to note that O_2_ concentrations in the atmosphere are much higher than CO_2_ concentrations, meaning that while oxygen equilibration remained efficient, CO_2_ exchange could have been substantially impacted by the lid. In addition, the bubbling applied for water mixing and aeration was intentionally very gentle in order to avoid disturbing the water column and the biological communities. While sufficient for its purpose, this low-intensity bubbling was not adequate to promote efficient CO_2_ equilibration. Further investigation might be necessary to fully understand the impact of the lid on CO_2_ exchange. Nevertheless, preventive measurements will be necessary in future experiments to avoid contamination of the oligotrophic seawater, ensuring the consistency of the data on marine biota collected by other research teams.

Figure 6 shows the behavior of Ω_ar_ and pCO_2_ calculated from the field and laboratory data. The observed variations align with the previously observed TA increases and pH decreases (see Figure 2). In all mesocosms, the initial pCO_2_ calculated from the field data was lower than the expected 420 ppm, likely due to restricted CO_2_ equilibration in the mesocosms. In contrast, the pCO_2_ calculated from the samples analyzed in the laboratory at Politecnico di Milano increased in a way that is consistent with the TA and DIC changes. In all cases, both L and H treatments exhibited lower pCO_2_ levels than the control, suggesting that the system would have absorbed more CO_2_ without the lids covering the mesocosms, over a period lasting several days or possibly weeks. These lids were used to prevent contamination from external sources, which was particularly important for the biological measurements, although they may have reduced the efficiency of CO_2_ equilibration with the atmosphere. Oxygen exchange was likely less affected by the presence of the lids because atmospheric O_2_ concentrations are much higher than those of CO_2_. Increasing the air–water interface of the mesocosms could further improve the gas exchange efficiency in future experiments.

**Figure 6:**
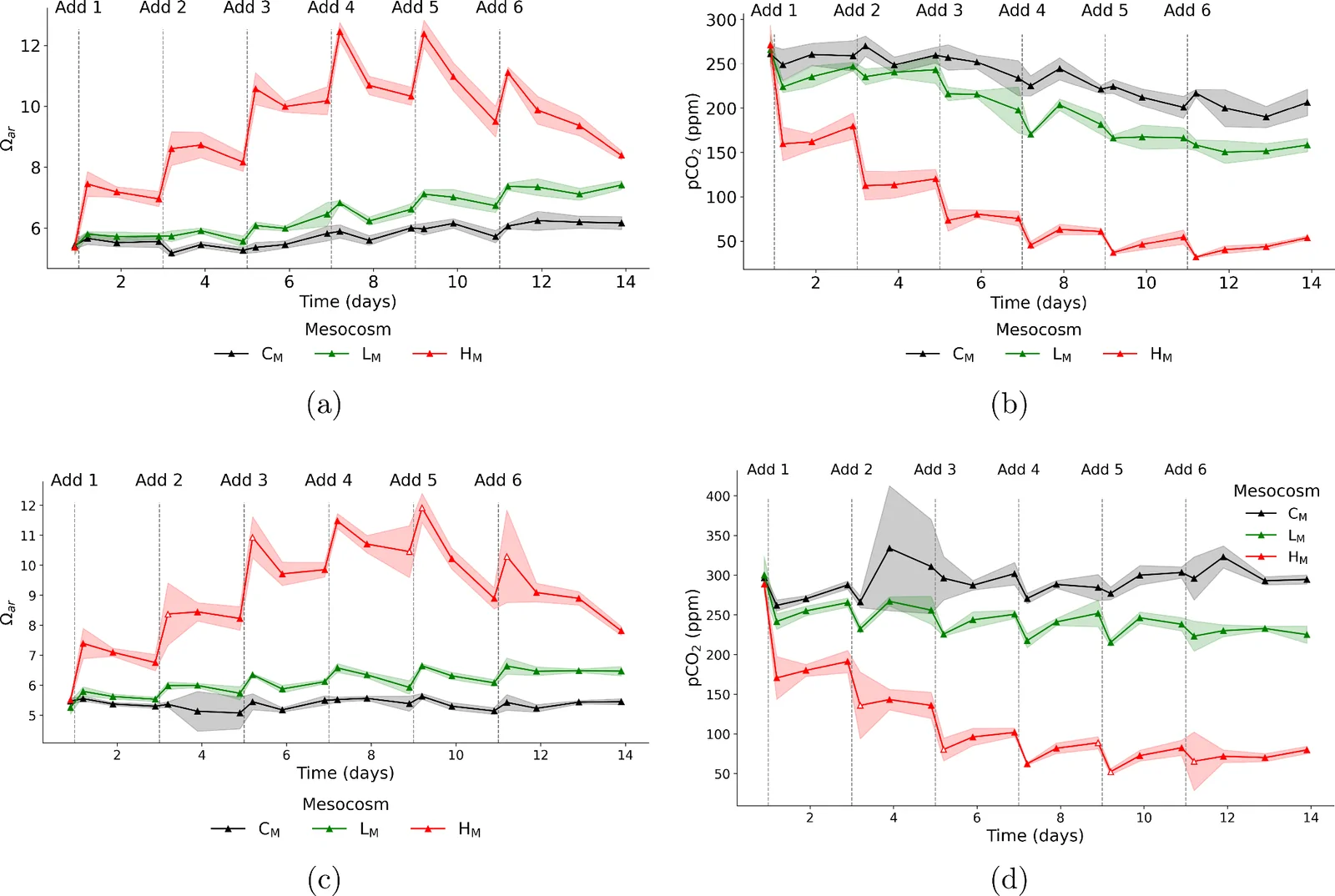
Evolution of Ω_ar_ and pCO_2_, calculated from (a, b) field measurements and (c, d) measurements on delivered samples. Vertical lines signal the additions of Ca­(OH)_2_, and white symbols indicate points with large standard deviation of the mean.

To gain more insight, we also analyzed the sediments collected at the bottom of the mesocosm. Although some precipitation was visually observed in the L treatment, the amount of material collected at the end of the experiment was insufficient for XRD analysis. Therefore, only samples from H treatment were examined by this technique.

A total of three mineral phases were identified: aragonite, calcite, and halite. Excluding halite (NaCl), which is likely formed during drying of the samples, the other phases are carbonates. Aragonite was the dominant carbonate phase in all samples, accounting for 85–92% of the identified mineral content, whereas calcite was present only in minor proportions (2–5%). A small number of diffraction peaks remain unexplained, particularly in sample H2 (see the SI); however, their low intensity suggests the presence of additional mineral phases only in trace amounts. These phases could not be unambiguously identified and were therefore excluded from the quantitative analysis.

Interestingly, no brucite (Mg­(OH)_2_) was detected. This is noteworthy, as in previous investigations, we detected its occurrence in both natural and artificial seawater.^22^ It is likely that magnesium hydroxide formed within the high-concentration slurries but redissolved upon dilution in the mesocosms, as suggested by Ringham et al.^15^

Raman spectroscopy was performed on the precipitates collected from all mesocosms. The repartition of the detected minerals is presented in Figure 8. In the C mesocosms, the precipitate contained aragonite, calcite, and other minerals in varying proportions, including huntite (CaMg_3_(CO_3_)_4_), cerussite (PbCO_3_), and terrigenous minerals such as quartz, K-feldspar, plagioclase, muscovite, pyrite, magnetite, hematite, and titanite. In the L and H mesocosms, the spectra confirmed the XRD results showing a dominant presence of aragonite with smaller amounts of calcite, while the minerals detected in the control were not detected (see Figure 7 and the Supporting Information). No halite was detected, likely because of methodological limitations and the selection of minerals targeted for analysis.

**Figure 7:**
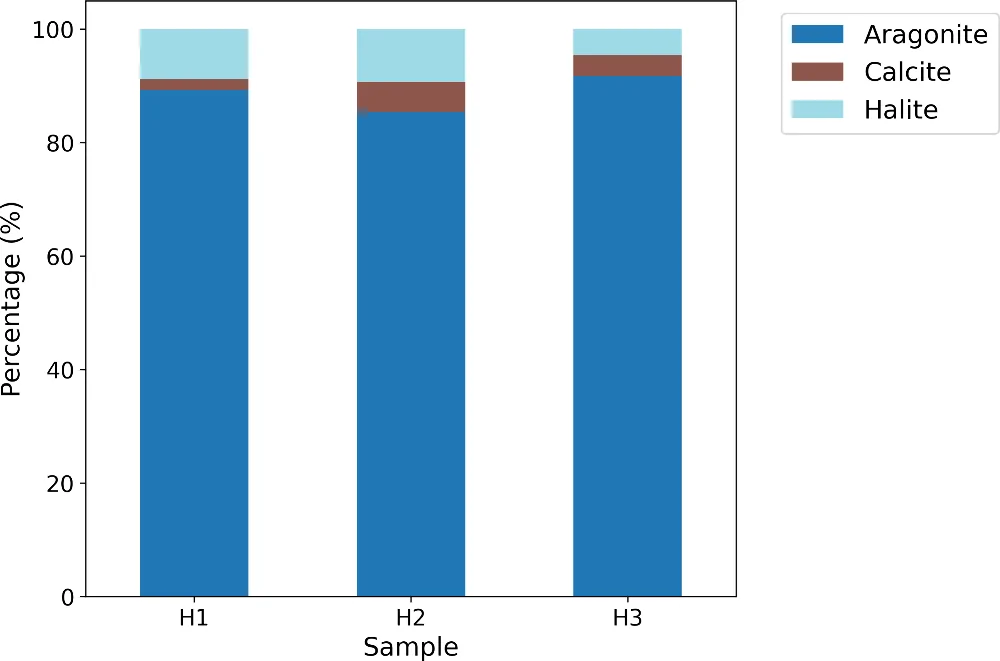
Repartition of minerals identified in the XRD analysis from the mesocosms H.

**Figure 8:**
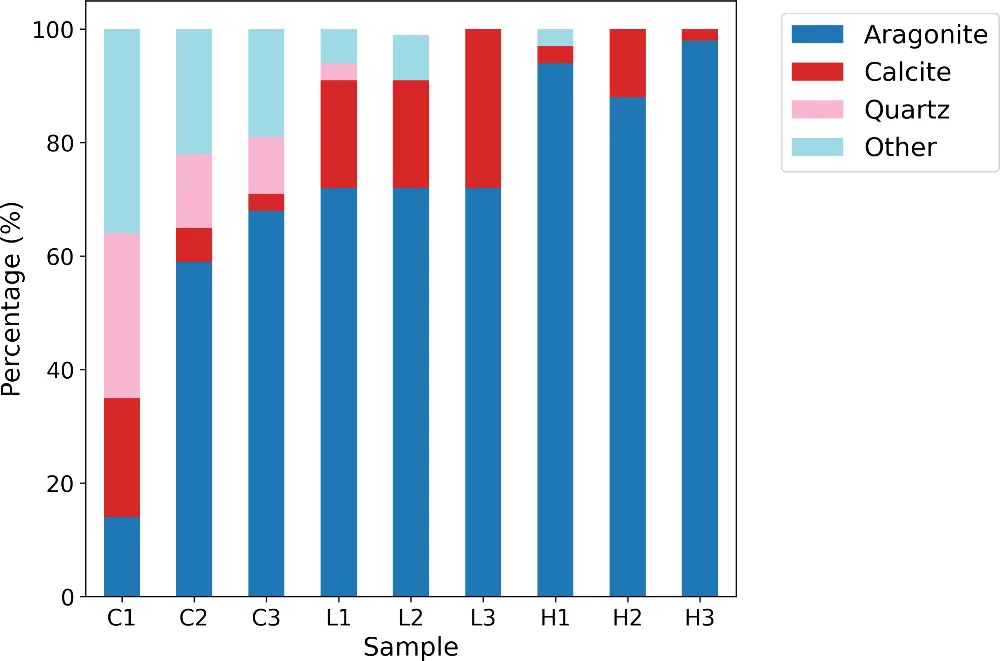
Repartition of minerals identified in the Raman spectra from the different mesocosms. The category “Other” includes huntite, cerussite, quartz, K-feldspar, plagioclase, muscovite, pyrite, magnetite, hematite, and titanite.

## Conclusion

We have carried out an experiment intended to test the effects of enhanced alkalinization induced by liming. Studies were carried out at the Hellenic Centre for Marine Research (HCMR) in Heraklion (Crete, Greece), using mesocosms. A fundamental knowledge gap was the safe quantity of SL that can be added without triggering the unwanted precipitation of CaCO_3_, which implies a loss of efficiency of the liming process. The results of our experiment enabled us to assess that, for the specific conditions of Crete seawater, an SL addition corresponding to 120 μmol L^–1^ TA fulfills the “safety criterion”. Of course, this threshold cannot be considered universally valid because different seawater conditions and methods of Ca­(OH)_2_ addition could generate localized pH spikes, potentially causing the precipitation of CaCO_3_ or even Mg­(OH)_2_.

Our experiment was not designed to monitor and optimize the uptake of atmospheric CO_2_ by the treated seawater, but the results reported here imply that it can be a slow process requiring several days or weeks, even when alkalinization is confined to a surface layer with a depth of a few meters. Further experimentation could help optimize delivery techniques for the alkaline plume, thereby enhancing the efficiency of CO_2_ uptake by seawater. In particular, future mesocosm designs should aim to improve air-sea gas exchange, avoiding complete covering of the systems and preserving the air–water interface area. Also, it would be worthwhile to plan on-site measurements of the DIC (such as the pH and alkalinity). This would complicate the logistics and increase the cost of the experiments but would improve the overall quality and internal consistency of the data. Once these limitations are overcome, the protocol we have proposed here for experiments and for calculation of the efficiency of OL could also be adopted at other locations, producing a geographical map of safe SL addition thresholds.

## Supplementary Material





## Notes

Raw experimental data and their elaboration (XLSX)
Additional data, including control charts, comparisons between measured and calculated TA, and XRD patterns (PDF)
