# Supplementary material for: Response of the Carbonate System to Ocean Alkalinity Enhancement: A Mesocosm-Scale Experiment
Source: ACS ES T Water. 2026 Sep 26;6(10):6316–24. doi: 10.1021/acsestwater.6c00342 (PMC13660394; doi:10.1021/acsestwater.6c00342)
Supplement: Supplementary file 1 [file ew6c00342_si_002.pdf]

Response of the carbonate system to Ocean Alkalinity Enhancement: a mesocosm-scale experiment

SUPPORTING INFORMATION

Selene Varliero,[†] Piero Macchi,[*,†] Daniela Basso,[‡] Paraskevi Pitta,[¶] Iordanis Magiopoulos,[¶] Chiara Santinelli,[§] Giovanni Checcucci,[§] Silvia Valsecchi,[‖] Laura Borromeo,[‡] and Guido Raos[*,†]

†*Department of Chemistry, Materials and Chemical Engineering "G. Natta", Politecnico di Milano, Via L. Mancinelli 7, I-20131 Milano, Italy*

‡*Department of Earth and Environmental Sciences, University of Milano-Bicocca, Piazza della Scienza 4, 20126 Milano, Italy*

¶*Institute of Oceanography, Hellenic Centre for Marine Research, P.O. Box 2214, 71003 Heraklion, Crete, Greece*

§*CNR-IBF, Istituto di Biofisica, Area della Ricerca, Via Moruzzi 1, I-56124 Pisa, Italy*

‖*Marine and Environmental Sciences Centre (MARE)/Aquatic Research Network (ARNET), Regional Agency for the Development of Research, Technology and Innovation (ARDITI), 9020-105 Funchal, Madeira Island, Portugal*

⊥*Faculty of Life Sciences, University of Madeira, 9000-072 Funchal, Madeira Island, Portugal*

E-mail: piero.macchi@polimi.it; guido.raos@polimi.it

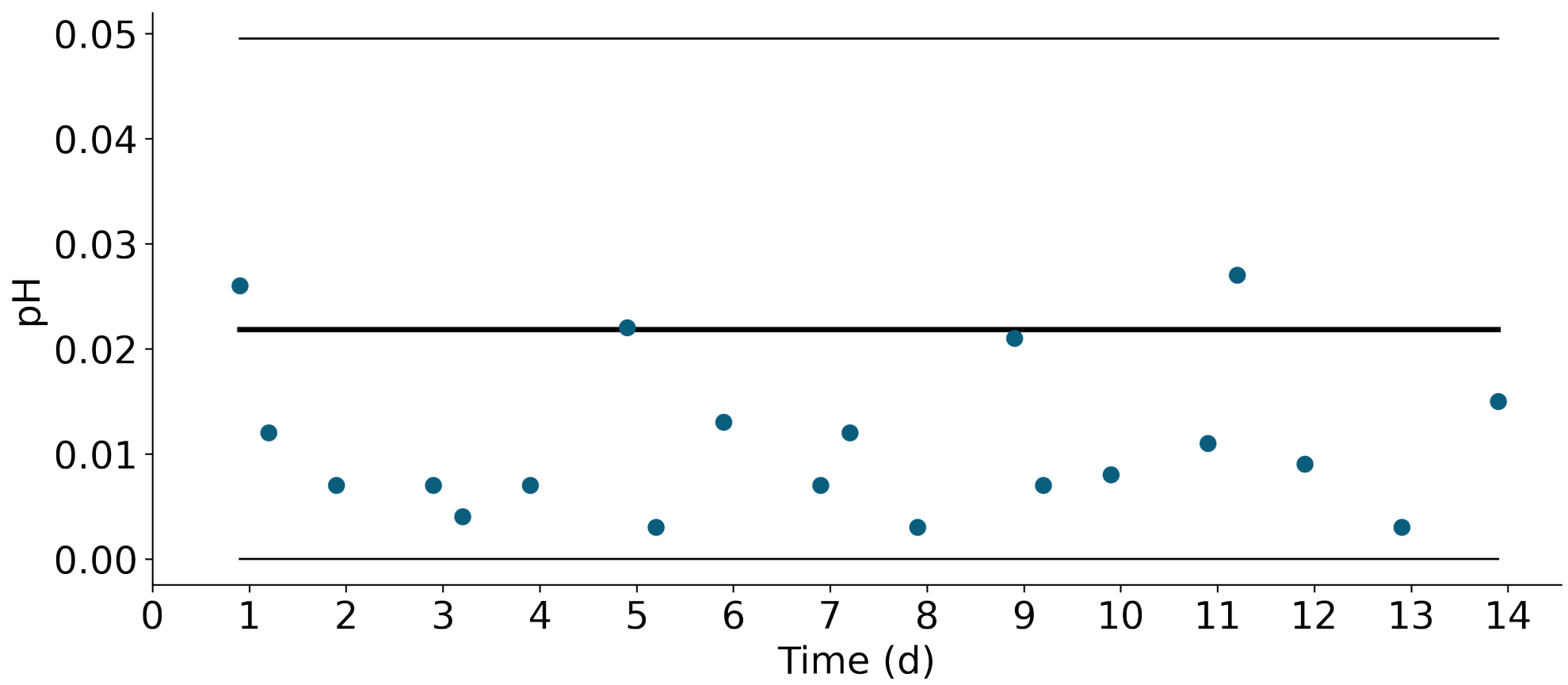

(a)

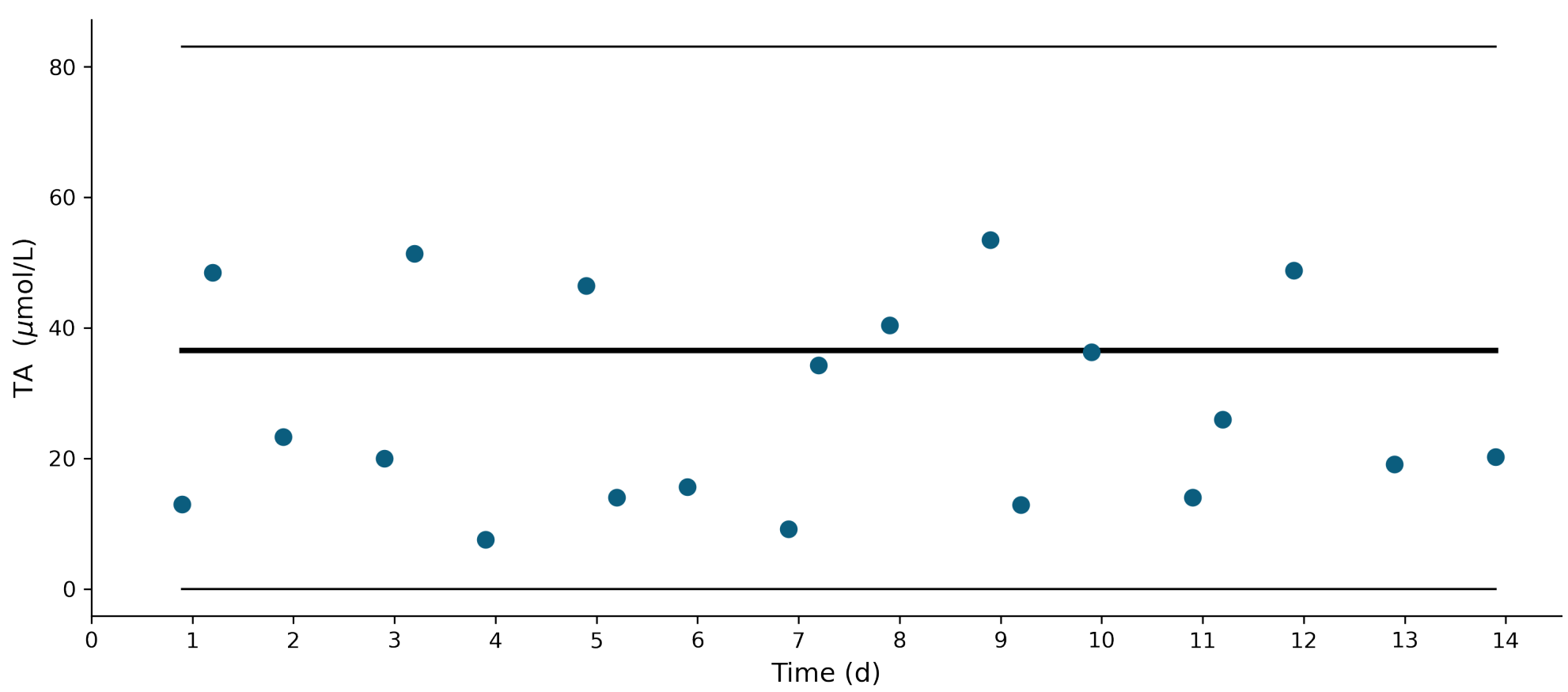

(b)

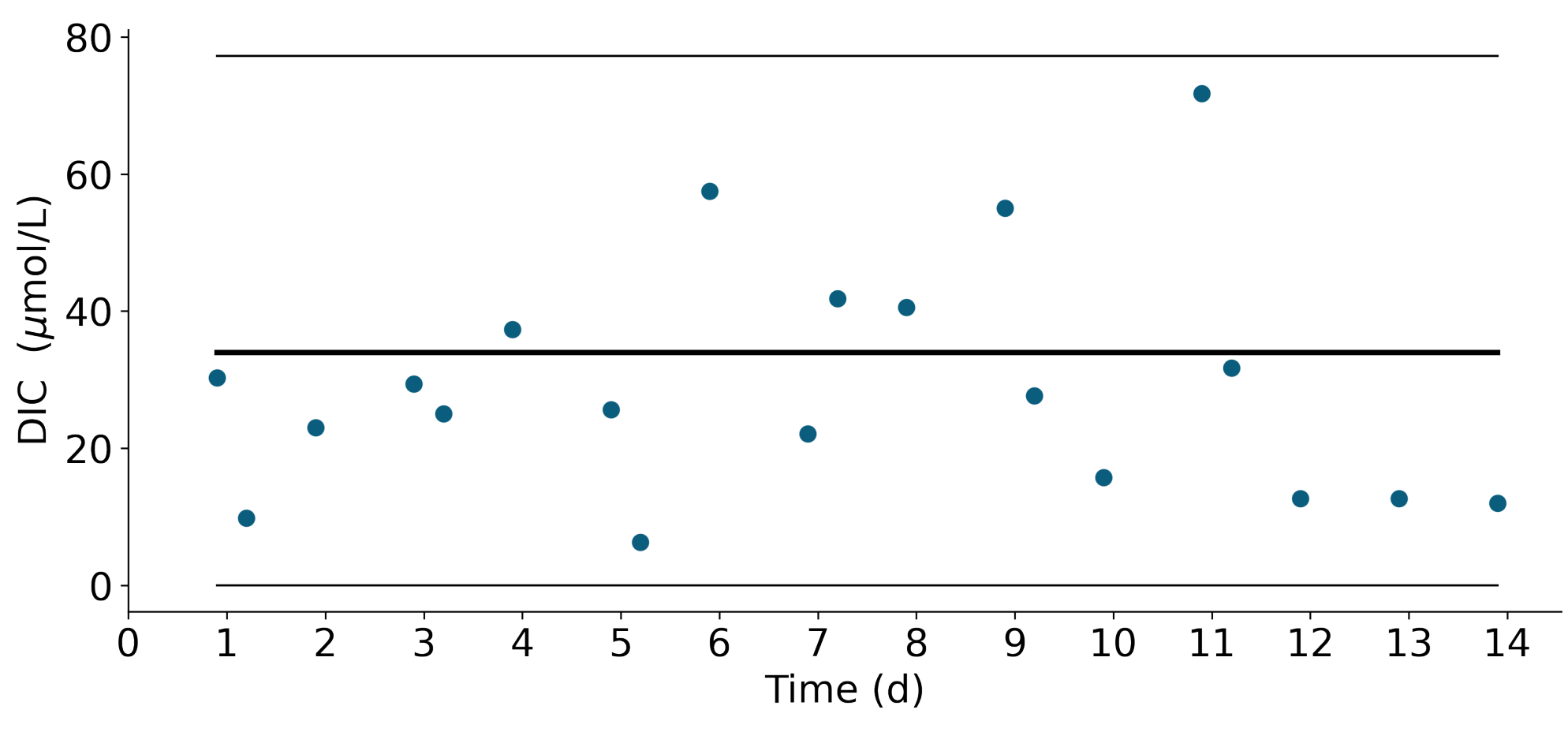

(c)

Figure S1: Control S-charts for the L mesocosms, showing the sample standard deviations of pH, TA, and DIC.

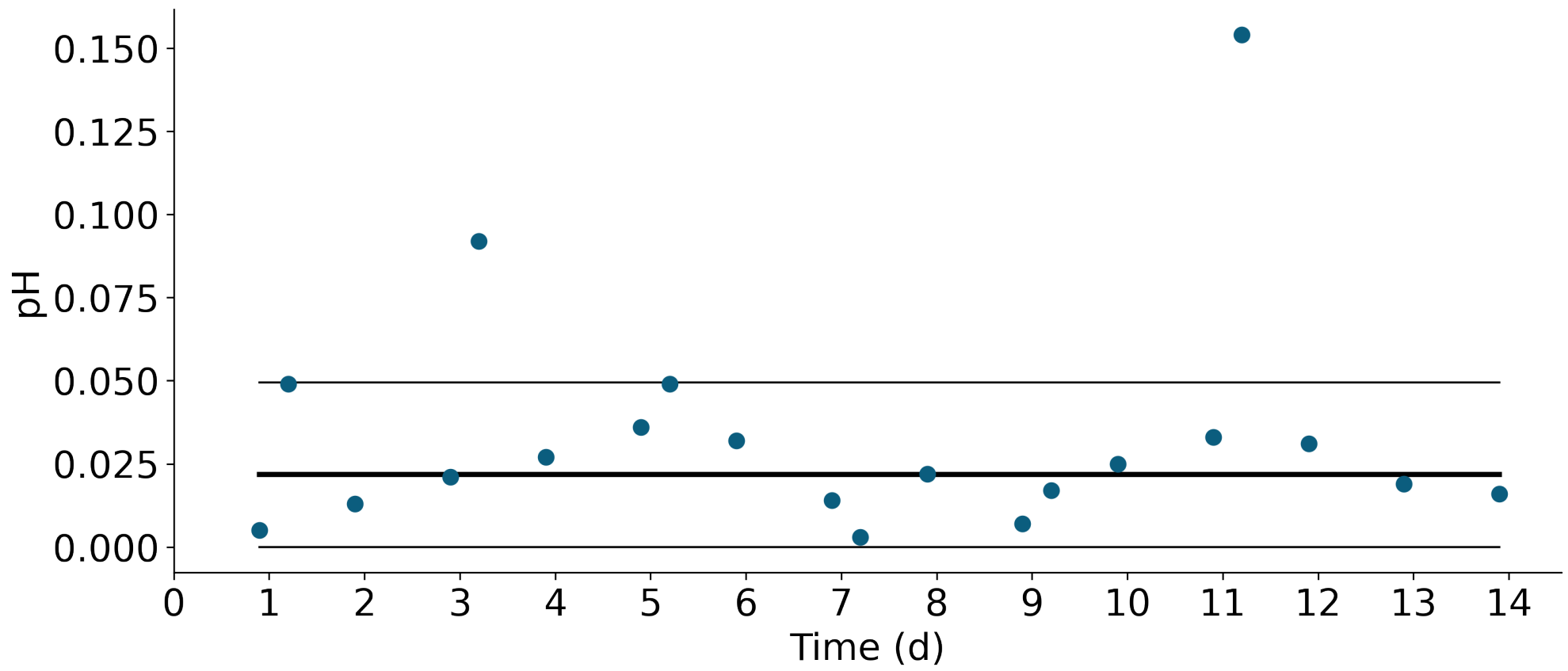

(a)

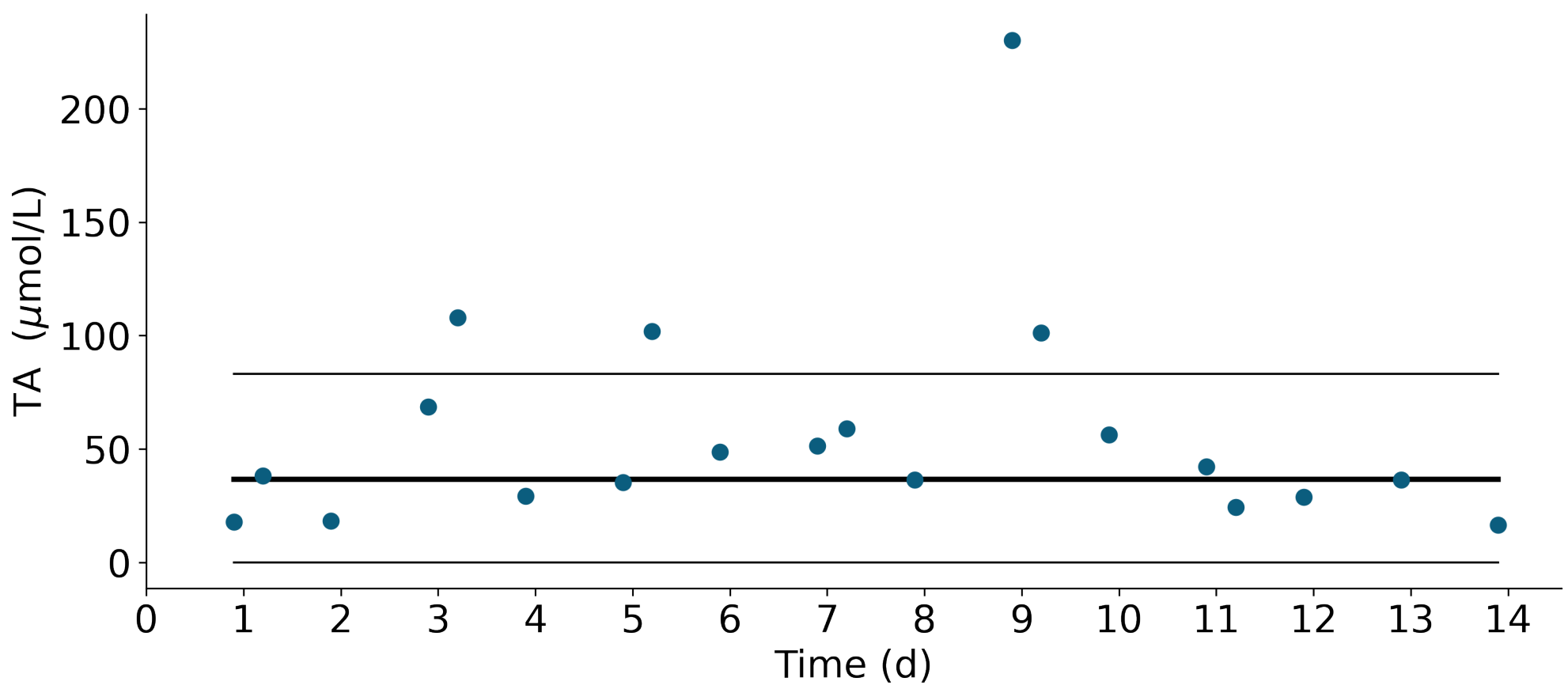

(b)

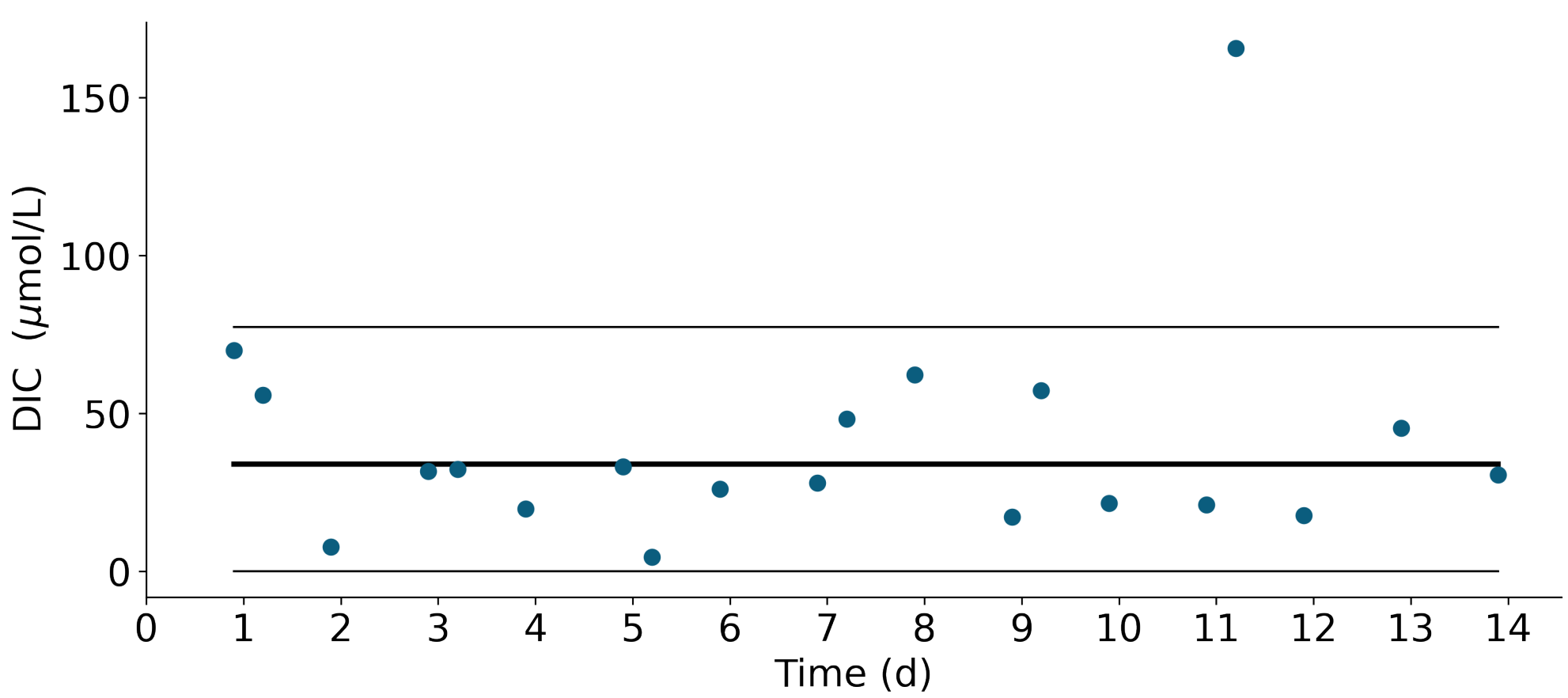

(c)

Figure S2: Control S-charts for the H mesocosms, showing the sample standard deviations of pH, TA, and DIC.

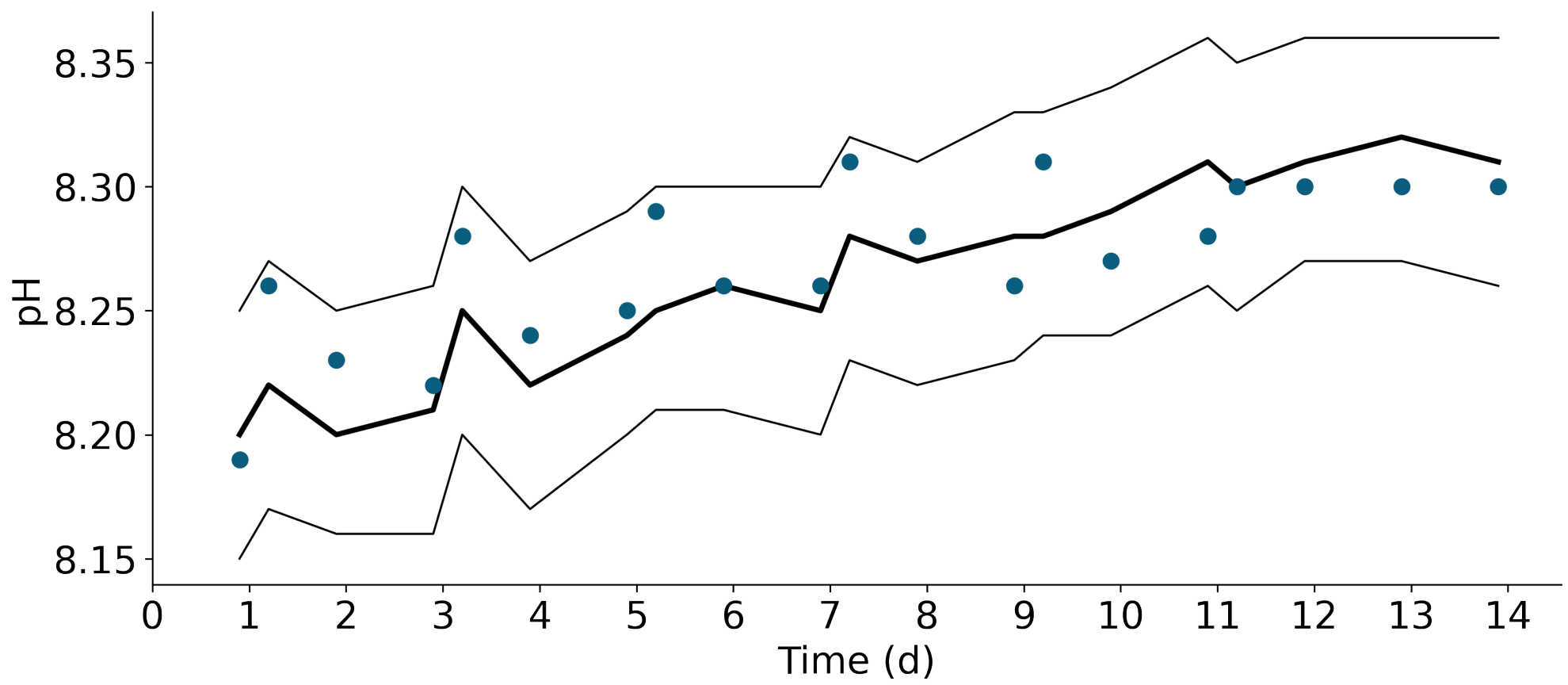

(a)

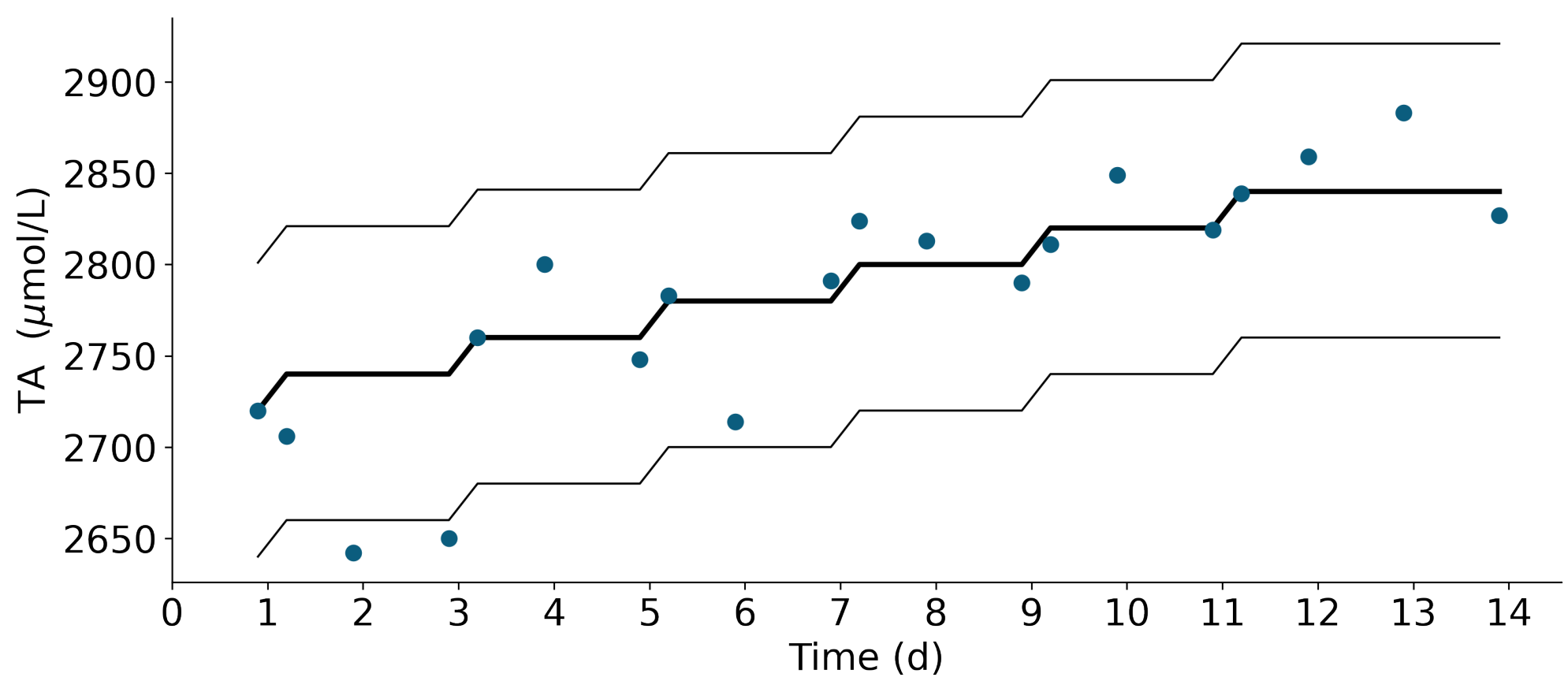

(b)

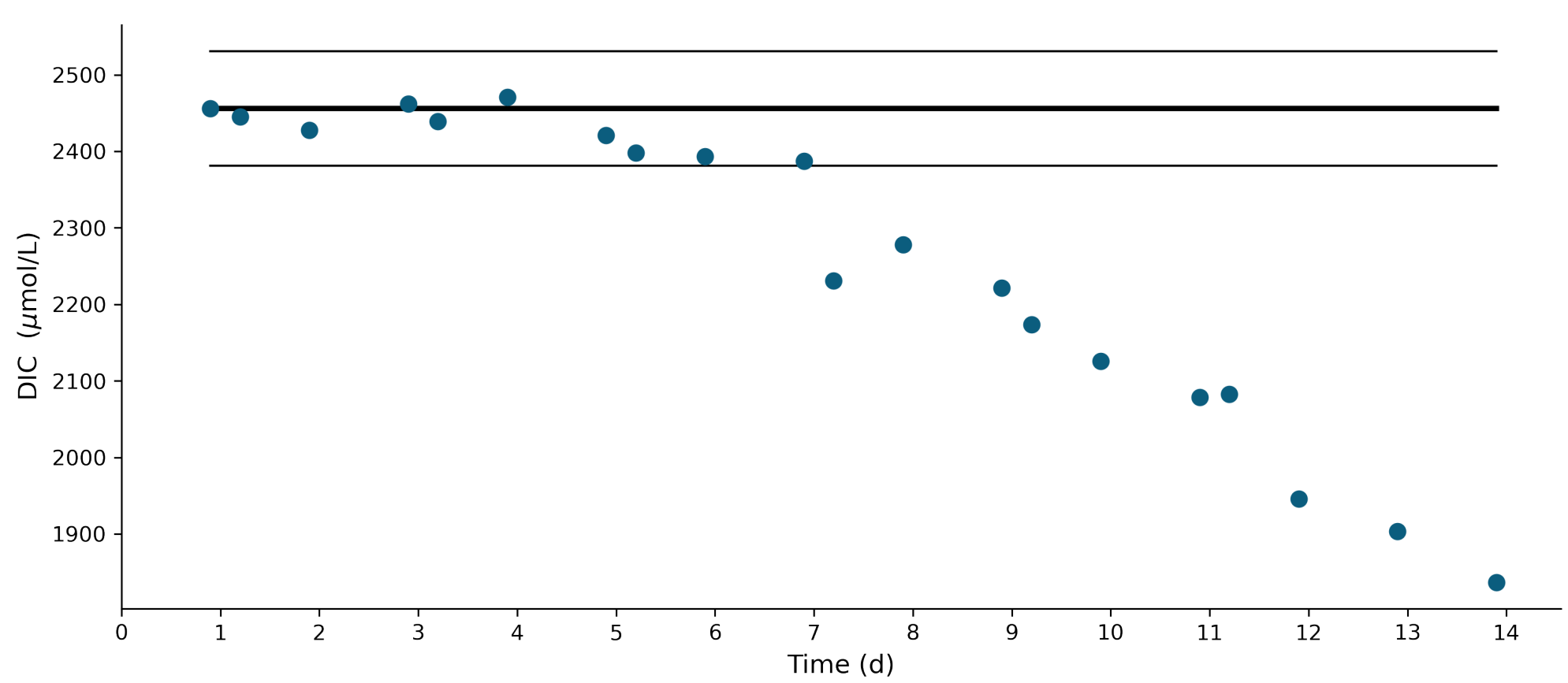

(c)

Figure S3: $\overline{X}$ Control charts for the L mesocosms, showing the temporal evolution of the sample mean pH, TA, and DIC values during the experiment.

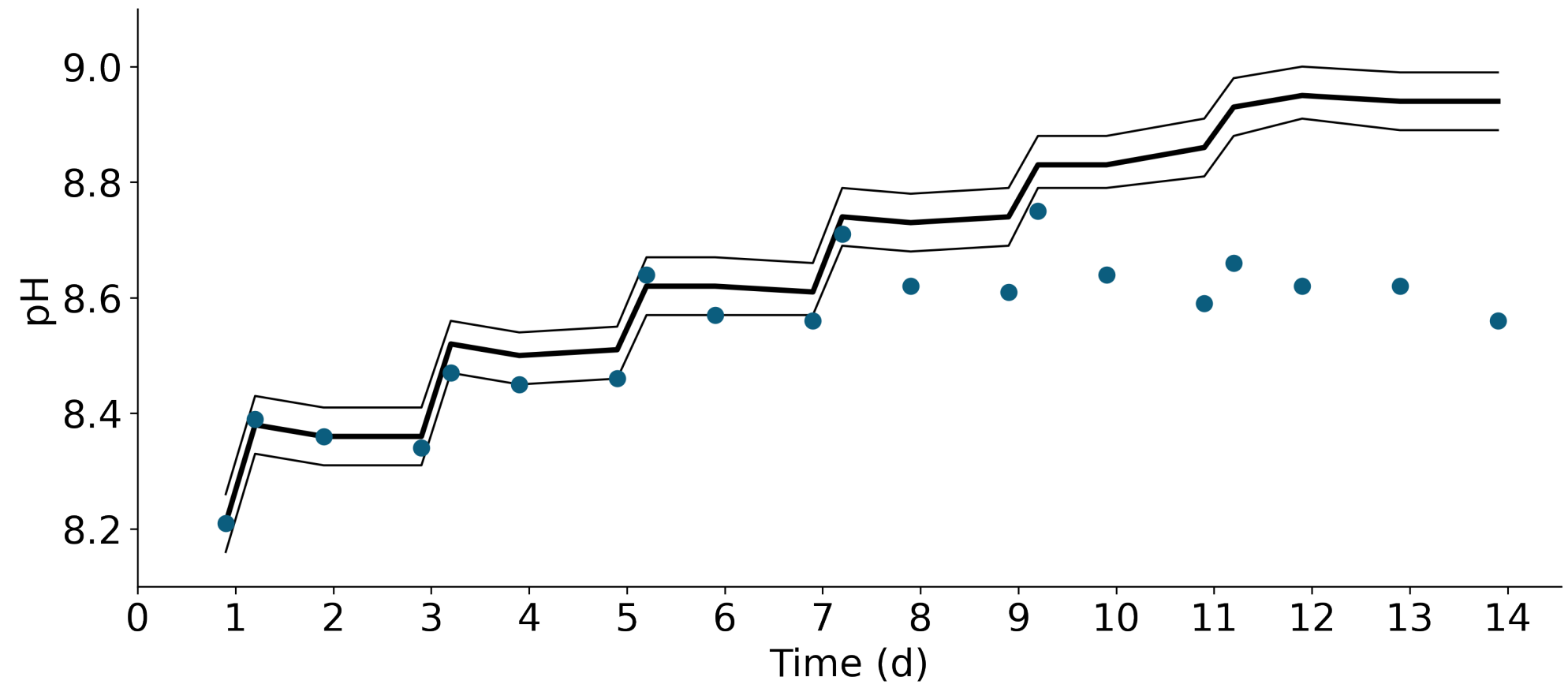

(a)

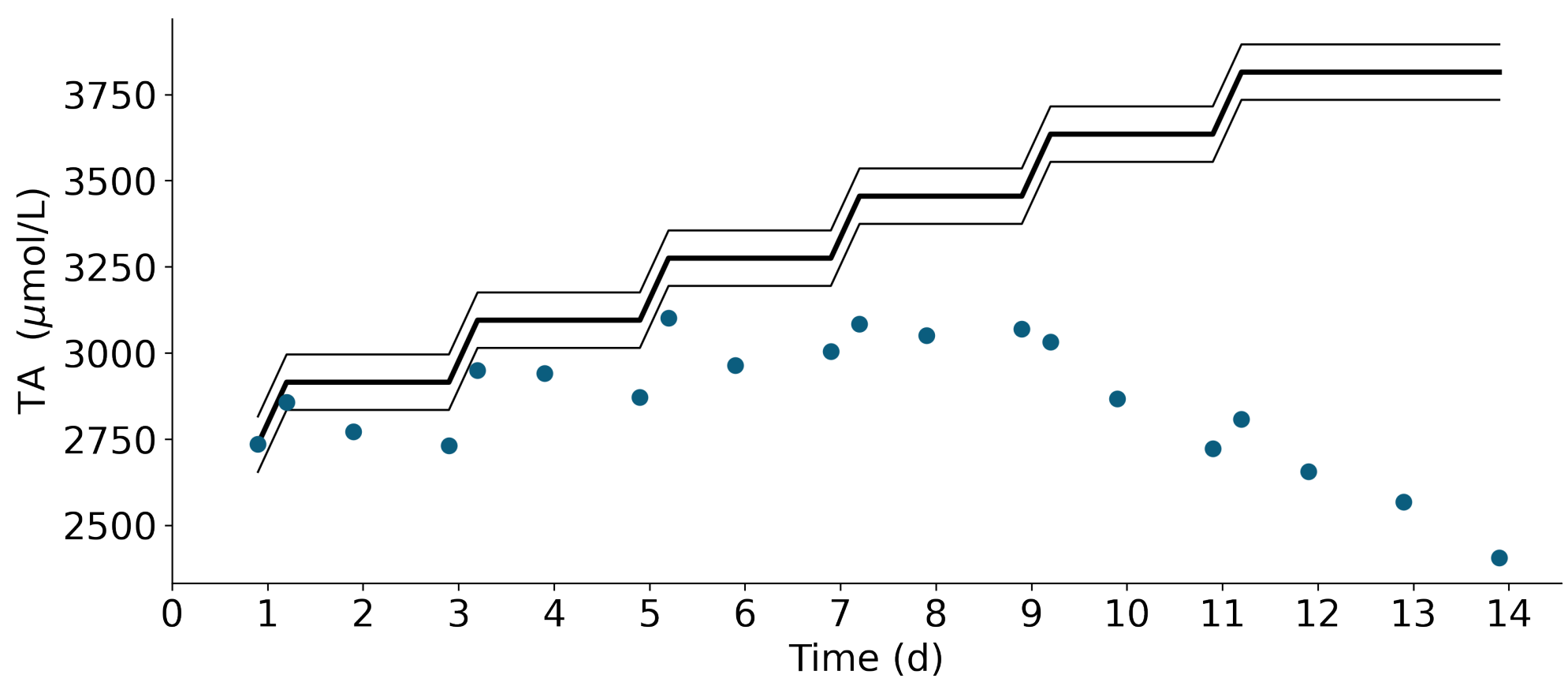

(b)

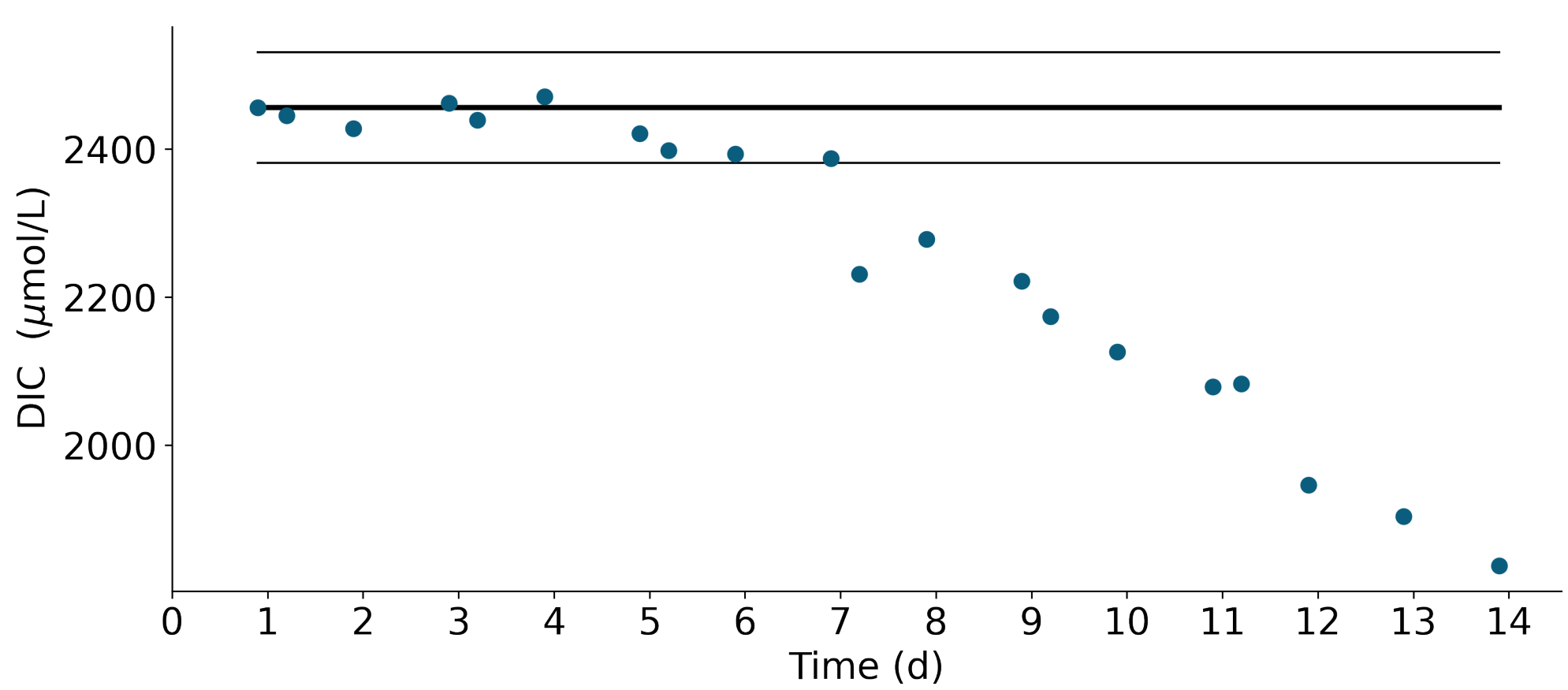

(c)

Figure S4: $\overline{X}$ Control charts for the H mesocosms, showing the temporal evolution of the sample mean pH, TA, and DIC values during the experiment.

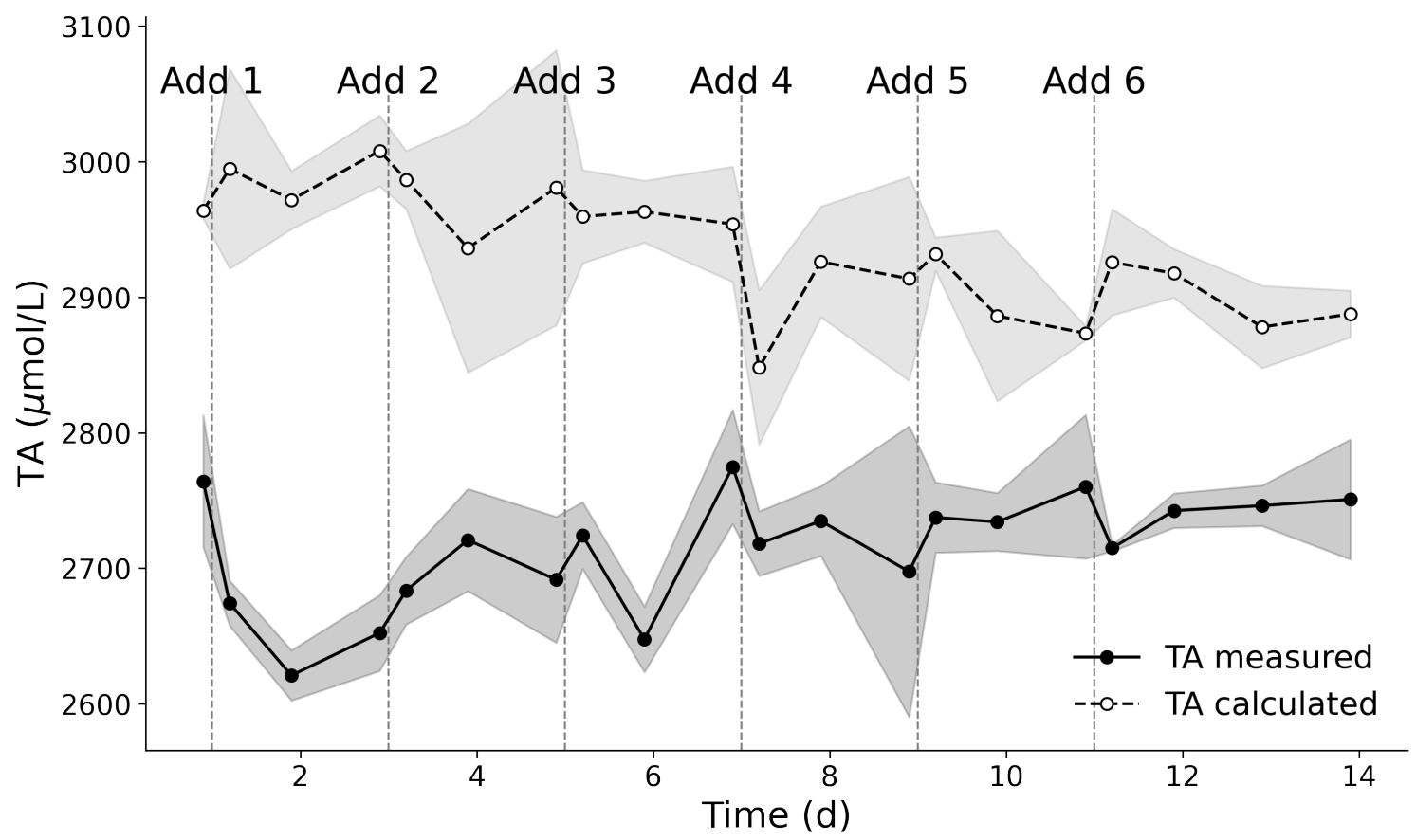

(a)

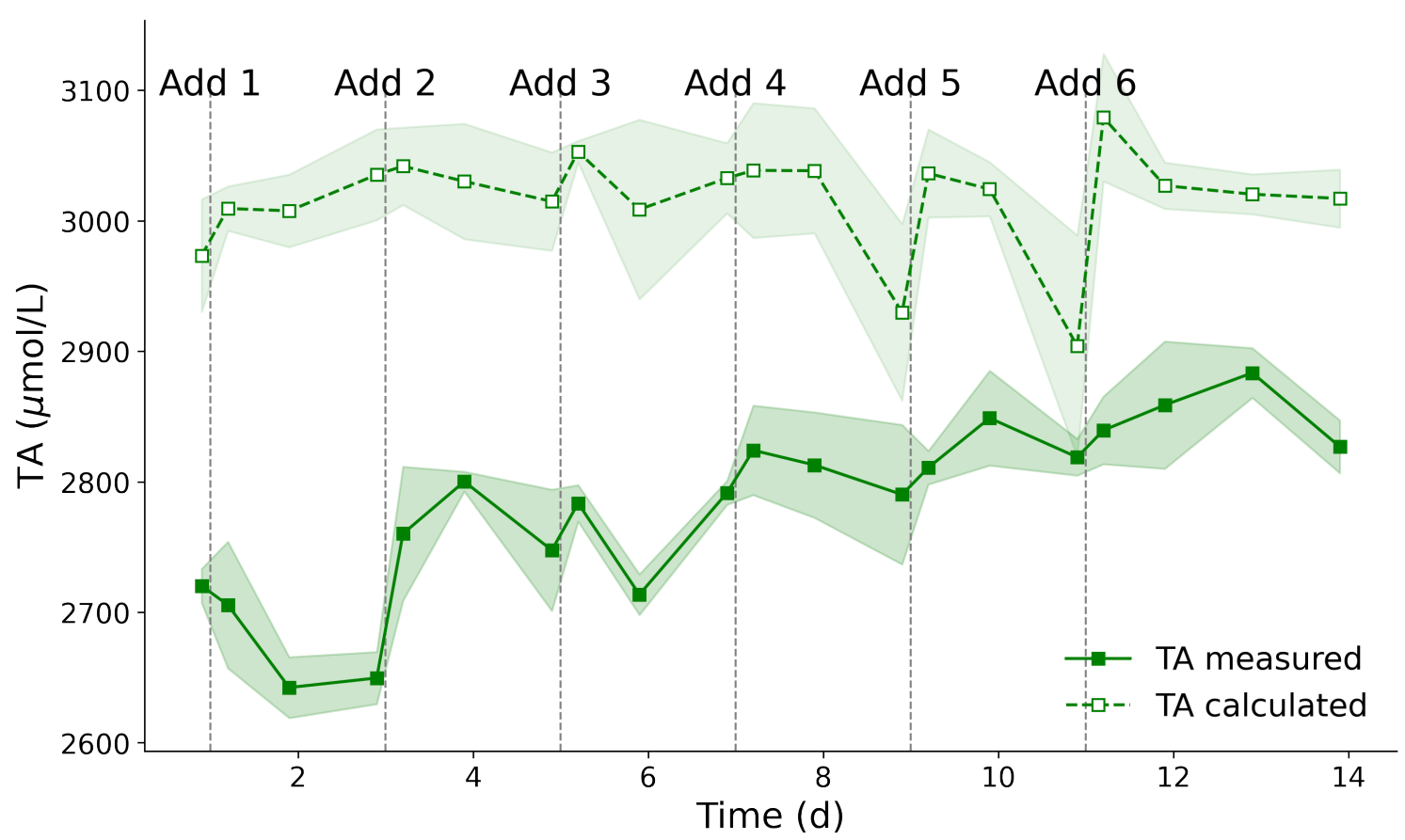

(b)

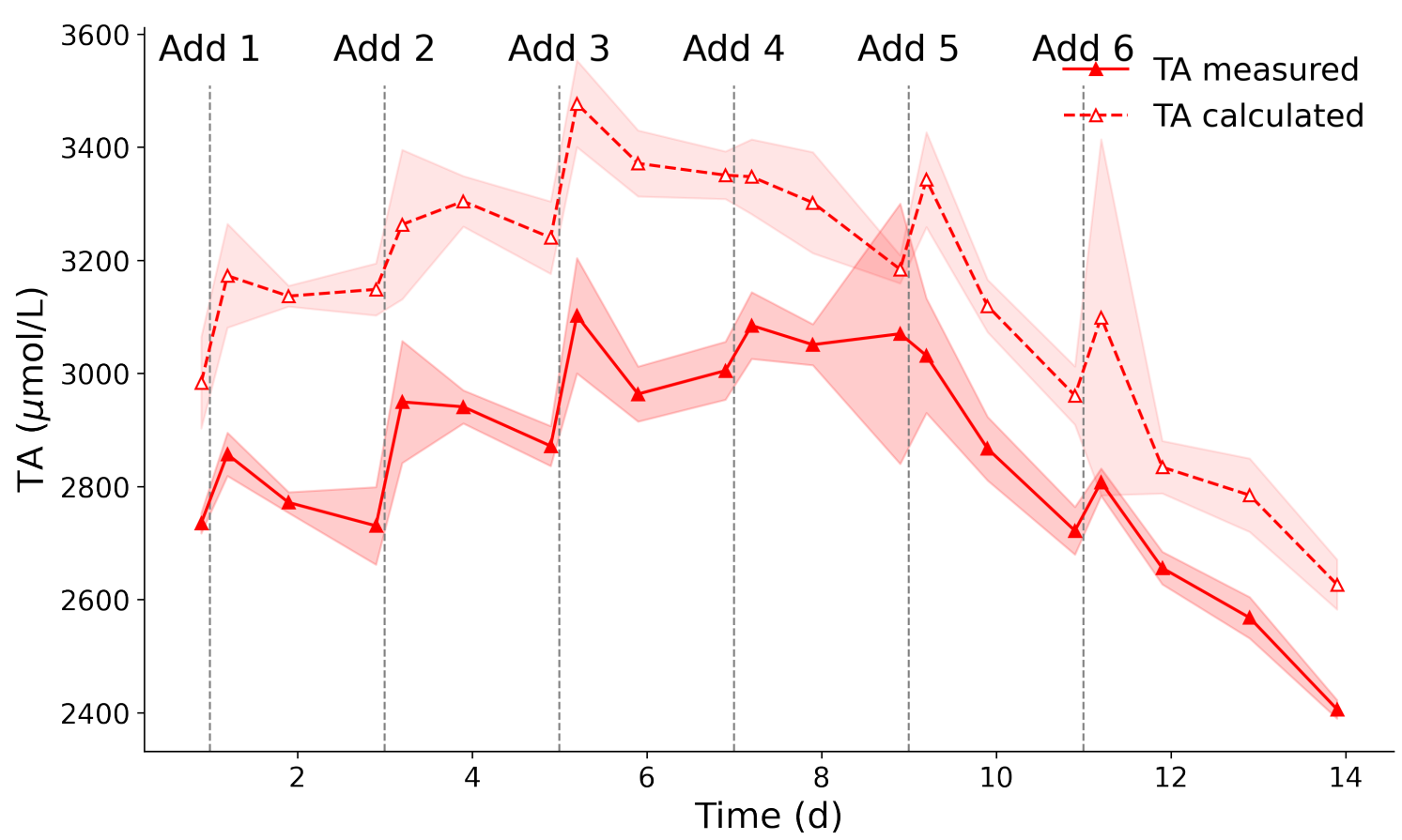

(c)

Figure S5: Comparison between measured and calculated TA for mesocosms C (a), L (b), and H (c). Measured TA corresponds to laboratory measurements, and calculated TA was obtained using PyCO2SYS from pH and DIC measurements. Shaded areas represent the standard deviation.

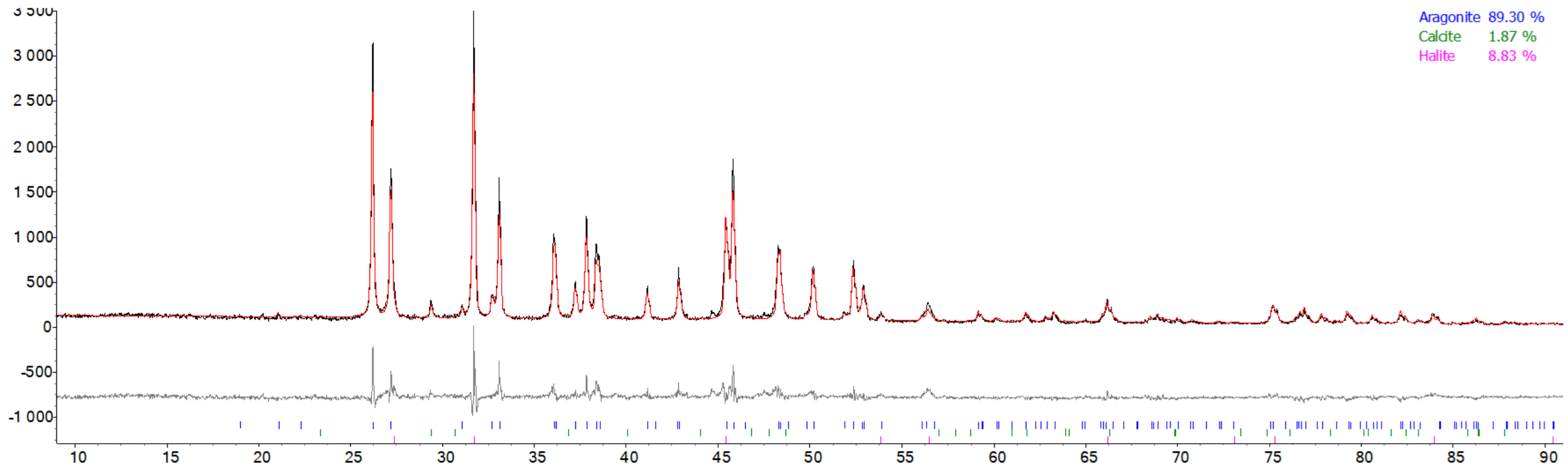

(a)

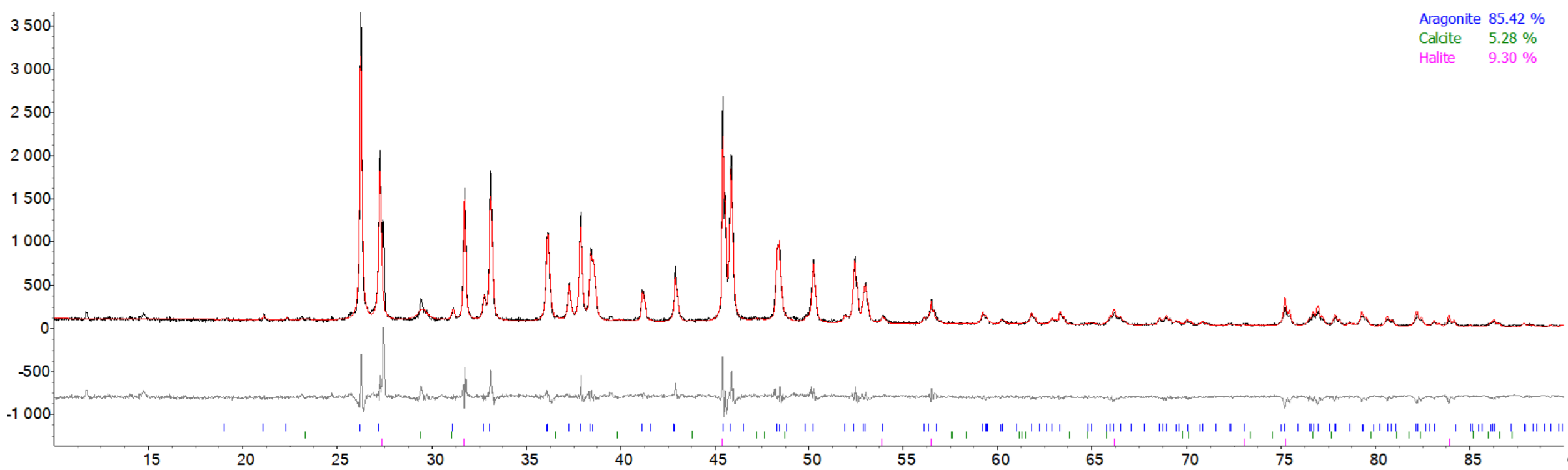

(b)

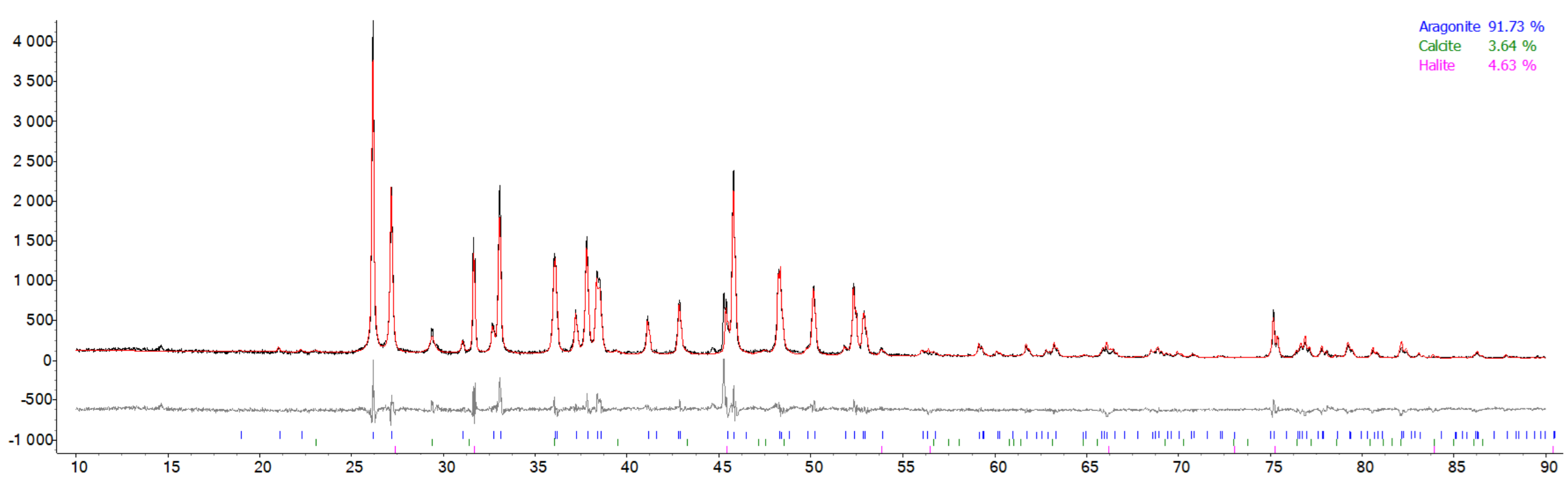

(c)

Figure S6: The XRD patterns collected on precipitates of the three mesocosms H: **(a)** H1, **(b)** H2, and **(c)** H3.
